# Multi-omics profiling identifies triciribine as a biomarker-guided antimetabolite therapy for acute lymphoblastic leukemia

**DOI:** 10.1038/s41698-026-01736-z

**Published:** 2026-10-14

**Authors:** Xuekang Qi, Georgios Mermelekas, Ondrej Hodek, Luay Aswad, Quentin Glaziou, Isabelle Rose Leo, Nona Struyf, Femke M. Hormann, Ioannis Siavelis, Henri Colyn Bwanika, Owen F. J. Hovey, Martin Haraldsson, Sean G. Rudd, Annika I. Johansson, Tom Erkers, Katja Pokrovskaja Tamm, Janne Lehtiö, Rozbeh Jafari

**Affiliations:** 1 Clinical Proteomics Mass Spectrometry, Department of Oncology-Pathology, Karolinska Institutet, Science for Life Laboratory, Tomtebodavägen 23A, 171 65 Solna, Sweden; 2 Swedish Metabolomics Centre, Department of Forest Genetics and Plant Physiology, Swedish University of Agricultural Sciences, Umeå, Sweden; 3 Molecular Precision Medicine, Department of Oncology-Pathology, Karolinska Institutet, Science for Life Laboratory, Tomtebodavägen 23A, 171 65 Solna, Sweden; 4 Department of Oncology-Pathology, Karolinska Institutet, Science for Life Laboratory, Tomtebodavägen 23A, 171 65 Solna, Sweden; 5 Department of Oncology-Pathology, Karolinska Institutet, J6:140 BioClinicum, Akademiska stråket 1, 171 64 Solna, Sweden; 6 Chemical Biology Consortium Sweden, Science for Life Laboratory, Department of Medical Biochemistry and Biophysics, Karolinska Institutet, 171 65 Stockholm, Sweden; 7 Swedish Metabolomics Centre, Department of Plant Physiology, Umeå University, Umeå, Sweden

**Keywords:** Cancer, Drug discovery, Oncology

## Abstract

Acute lymphoblastic leukemia (ALL) exhibits molecular and metabolic heterogeneity that limits therapeutic efficacy. Although antimetabolites remain central to ALL treatment, determinants of therapeutic sensitivity and resistance remain incompletely understood. Here, we investigated the activity and molecular response landscape of the purine analog triciribine (TCN) across diverse ALL models using integrated phosphoproteomics, thermal proteome profiling, and quantitative proteomics. TCN showed potent cytotoxicity across ALL cell lines and a drug response profile distinct from canonical PI3K–Akt–mTOR inhibitors. Early phosphoproteomics revealed no consistent suppression of Akt pathway signaling, with transient activation of several Akt-associated readouts and inhibition of cyclin-dependent kinase signaling. The monophosphorylated metabolite TCN-P was the predominant intracellular active species, and adenosine kinase (ADK) was required for TCN-P generation and cytotoxicity. Proteome Integral Solubility Alteration profiling identified candidate TCN-P interactions involving nucleotide metabolism, DNA replication, and protein synthesis, associated with impaired purine biosynthesis, DNA damage, translational stress, and cell-cycle perturbation. Across ALL cell lines and primary samples, ADK levels correlated positively with TCN sensitivity, supporting ADK-dependent metabolism as a major determinant of therapeutic response. Together, these findings identify TCN as a metabolically activated antileukemic agent with broad antimetabolite activity and nominate ADK as a potential predictive biomarker for precision therapy in ALL.

## Introduction

ALL is an aggressive hematologic malignancy that affects patients of all ages and is characterized by rapid proliferation and disease progression^1,2^. Extensive genetic and epigenetic heterogeneity underlies substantial diversity in molecular features, cellular behavior, and clinical responses to therapy. Although this complexity complicates the development of universally effective targeted treatments, leukemic cells remain uniformly dependent on high biosynthetic and metabolic flux, creating shared vulnerabilities across ALL subtypes. Altered cellular metabolism, therefore, represents not only a hallmark of ALL but also a functional dependency that may be less constrained by genotype^3,4^.

Nucleoside and nucleotide metabolism constitutes a fundamental requirement for leukemic cell survival and proliferation. Rapidly proliferating leukemic cells require sustained nucleotide synthesis to support DNA replication, RNA transcription, protein translation, and energy homeostasis, rendering these pathways particularly sensitive to pharmacologic perturbation^5,6^. Disruption of these pathways has therefore been extensively exploited for therapeutic purposes^4,7^. Nucleoside analogs (NAs) interfere with nucleotide metabolism by targeting key enzymatic steps or by mimicking endogenous nucleosides, thereby impairing nucleic acid synthesis and inducing cytotoxic stress in rapidly proliferating cells^4,8,9^. Such agents have demonstrated substantial clinical activity in multiple malignancies, including ALL.

Despite major advances in risk stratification and targeted therapy, the backbone of ALL treatment remains broad cytotoxic chemotherapy, including several nucleoside analogs and antimetabolites^1,7,10^. While these agents have enabled high cure rates, predictive biomarkers that inform patient-specific sensitivity or resistance remain limited^1,7,9^. As a result, response variability and treatment-related toxicity continue to pose significant challenges, particularly in relapsed or refractory disease. Identifying molecular features that govern sensitivity to cytotoxic and antimetabolite therapies, therefore, represents an important unmet need, both to refine existing treatment strategies and to guide the development of next-generation agents.

TCN was originally developed as an adenosine analog, with its monophosphorylated form TCN-P representing the predominant intracellular and biologically active species^11^. Unlike many nucleoside analogs that are converted to active di- or triphosphate metabolites and/or exert activity through nucleic-acid incorporation, TCN is unusual in that its monophosphorylated form is sufficient for biological activity, suggesting alternative modes of action^8,12,13^. In clinical formulations, TCN-P functions as a water-soluble prodrug that is dephosphorylated extracellularly by phosphatases and ecto-phosphatases prior to cellular uptake^11^. Once inside the cell, TCN is re-phosphorylated by ADK, a step that is required for its cytotoxic activity^11,14^. Consistent with this requirement, ADK level has been associated with TCN sensitivity in multiple cellular contexts^15,16^.

Despite its classification as a nucleoside analog, TCN has frequently been described as a direct inhibitor of Akt signaling^17–19^. However, this model has failed to fully account for TCN’s cellular activity, which often exceeds that of canonical PI3K–Akt–mTOR pathway inhibitors^20^. In addition, TCN has been associated with inhibition of DNA synthesis, RNA synthesis, protein synthesis, and perturbation of purine nucleotide biosynthesis, indicating that its cellular effects may extend beyond a single signaling axis^12,13,21–23^. The limited predictive value of Akt pathway activation in clinical trials further underscores the incomplete understanding of TCN’s mechanism of action^24^.

Together, these observations position TCN as a mechanistically anomalous compound that does not conform neatly to established categories of either classical nucleoside analogs or targeted kinase inhibitors. Given this ambiguity, an unbiased and systems-level approach is required to define the molecular processes and pathways that govern TCN response. Proteomics based on liquid chromatography–mass spectrometry (LC–MS) provides a powerful platform to examine changes in protein abundance, post-translational modifications, proteoform diversity, and protein–ligand interactions at a global scale^25–28^. In this study, we applied complementary proteomic strategies, including thermal proteome profiling approaches such as proteome integral solubility alteration (PISA), quantitative time-course proteomics, and phosphoproteomics, to characterize cellular responses to TCN treatment in ALL models^27,29–31^. Rather than seeking a single dominant target, these approaches were used to identify the pathways and cellular processes associated with TCN sensitivity and response, providing a framework for mechanistic interpretation and biomarker development for translational applications.

## Results

### Triciribine exhibits antileukemic activity and a distinct response profile compared with Akt inhibitors

**Fig. 1:**
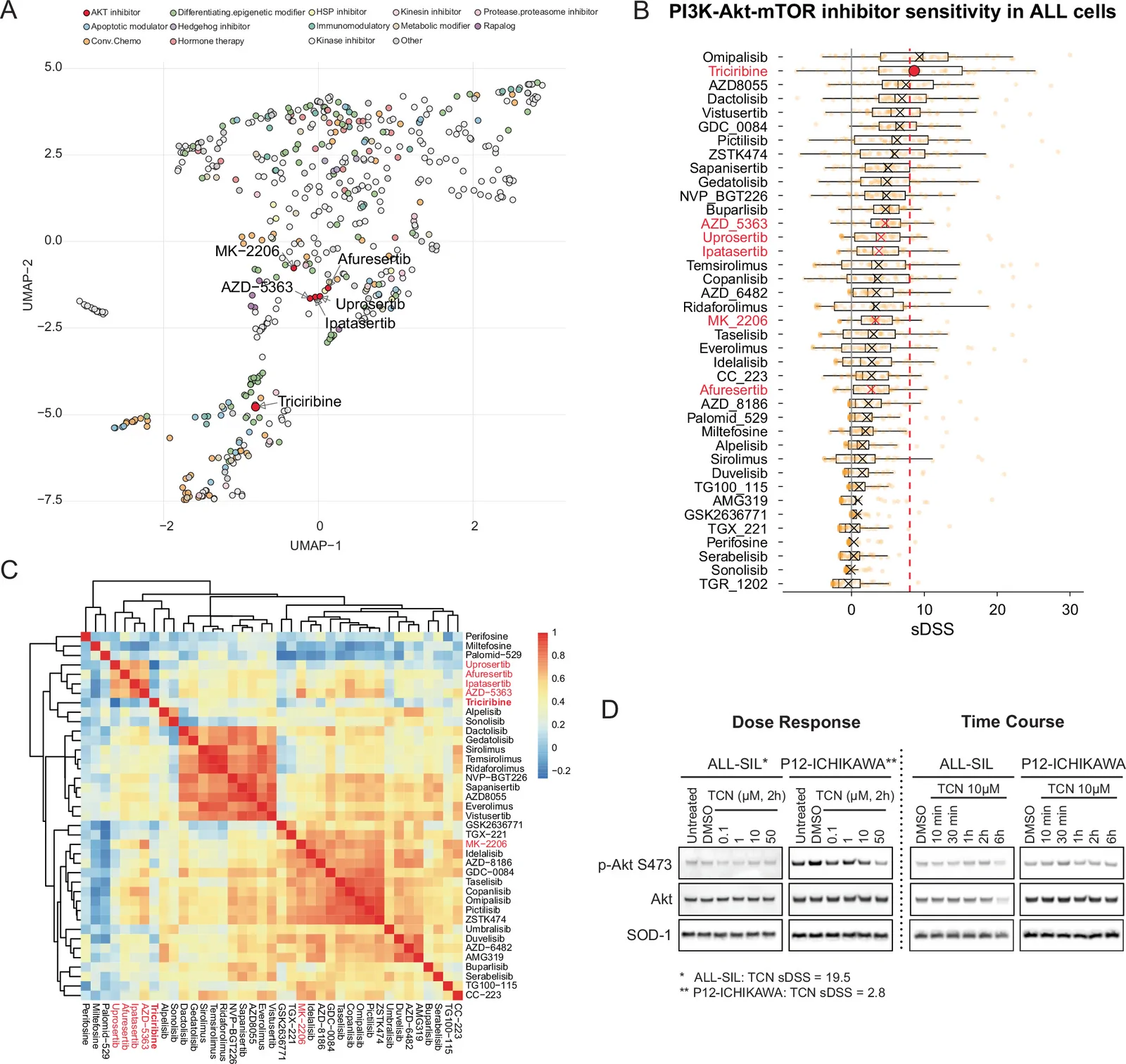
Drug sensitivity profiling of triciribine across ALL cell lines. **A** UMAP visualization of drug sensitivity profiles for 528 compounds tested across 43 ALL cell lines. Each point represents a compound, positioned based on the similarity of killing profiles. Data were obtained from the FORALL database. **B** sDSS values for TCN and PI3K–Akt–mTOR pathway inhibitors across 43 ALL cell lines. The dashed line indicates sDSS = 8. Boxes indicate the median and interquartile range (25th–75th percentile). Whiskers extend to the most extreme observations within 1.5× the interquartile range (Tukey method). Individual cell line responses are shown as orange dots. The mean sDSS value for each inhibitor is indicated by an “×” or a larger dot. Akt inhibitors are indicated in red. Data were obtained from the FORALL database. **C** Pairwise Pearson correlation analysis of drug sensitivity profiles for PI3K–Akt–mTOR pathway inhibitors across 43 ALL cell lines from the FORALL database. **D** Immunoblot analysis of phosphorylated Akt (Ser473), total Akt, and the loading control SOD1 in ALL-SIL and P12-ICHIKAWA cells following TCN treatment with indicated concentrations (0–50 μM for 2 h) or durations (0–6 h).

In our previous integrative multi-omics and drug sensitivity profiling of 43 childhood ALL cell lines, we observed that TCN displayed a response profile distinct from canonical Akt inhibitors (Fig. 1A)^32,33^. Pairwise correlation analysis of drug sensitivity profiles revealed no correlation between TCN and afuresertib, ipatasertib, or uprosertib (Supplementary Fig. 1A), whereas these canonical Akt inhibitors showed robust mutual correlations (Supplementary Fig. 1B). Analysis of large-scale pharmacogenomic screening data from the PRISM repurposing dataset^34^ showed that the majority of ALL and AML cell lines exhibited TCN sensitivity above the median response across all cancer cell lines, suggesting relative sensitivity and potential selectivity toward these hematologic malignancies (Supplementary Fig. 1C). In our dataset, TCN induced cytotoxicity across the majority of ALL cell lines and subtypes compared with normal bone marrow samples, with a substantial fraction displaying hypersensitivity (selective drug sensitivity score, sDSS ≥ 8). As sDSS represents drug sensitivity normalized against responses in normal bone marrow controls, these values reflect relative leukemia-selective activity rather than drug potency alone. In contrast, most PI3K–Akt–mTOR inhibitors showed markedly lower activity, with sDSS values below this threshold in the majority of ALL models (Fig. 1B). Consistent with these findings, pairwise correlation and dimensionality reduction analyses demonstrated generally low similarity between TCN and other PI3K–Akt–mTOR inhibitors, with only modest correlations observed for selected PI3K or PI3K/mTOR inhibitors such as pictilisib (Pearson *r* = 0.40, *n* = 43) and GDC-0084 (Pearson *r* = 0.45, *n* = 43) (Fig. 1C).

Analysis of the PRISM drug repurposing dataset^34^, encompassing 4518 compounds tested across 578 cancer cell lines representing many lineages, further validated these observations. TCN again segregated from the PI3K–Akt–mTOR inhibitor cluster (Supplementary Fig. 1D), suggesting that its distinct response profile is neither restricted to hematologic malignancies nor specific to a single dataset. Several HDAC inhibitors showed moderate correlations with TCN sensitivity in the ALL dataset (Pearson *r* ≤ 0.54); however, this association was not reproduced in the PRISM dataset (Supplementary Fig. 1D), suggesting that it may represent an ALL context-dependent relationship that does not extend broadly across cancer types and warrants further focused investigation.

To further examine the relationship between TCN activity and Akt signaling, we analyzed the PTEN-deficient T-ALL cell line P12-ICHIKAWA, which exhibits constitutive Akt activation^35^. Despite this phenotype, P12-ICHIKAWA cells were not hypersensitive to TCN (sDSS = 2.8), and their sensitivity to canonical Akt inhibitors was comparable to that of other ALL models. Dose–response western blotting revealed that phosphorylation of Akt at Ser473 decreased only at higher TCN concentrations (Fig. 1D). Moreover, time-course analysis demonstrated transient Akt phosphorylation following TCN exposure in both P12-ICHIKAWA cells and the TCN-sensitive ALL-SIL cell line (sDSS = 19.5) (Fig. 1D).

Together, these data indicate that TCN exhibits selective antileukemic activity and a response profile distinct from that of canonical Akt inhibitors, suggesting that additional mechanisms may contribute to its cellular effects.

### Phosphoproteomic profiling reveals early inhibition of CDK activity and activation of mTOR and MAPK signaling following triciribine treatment

**Fig. 2:**
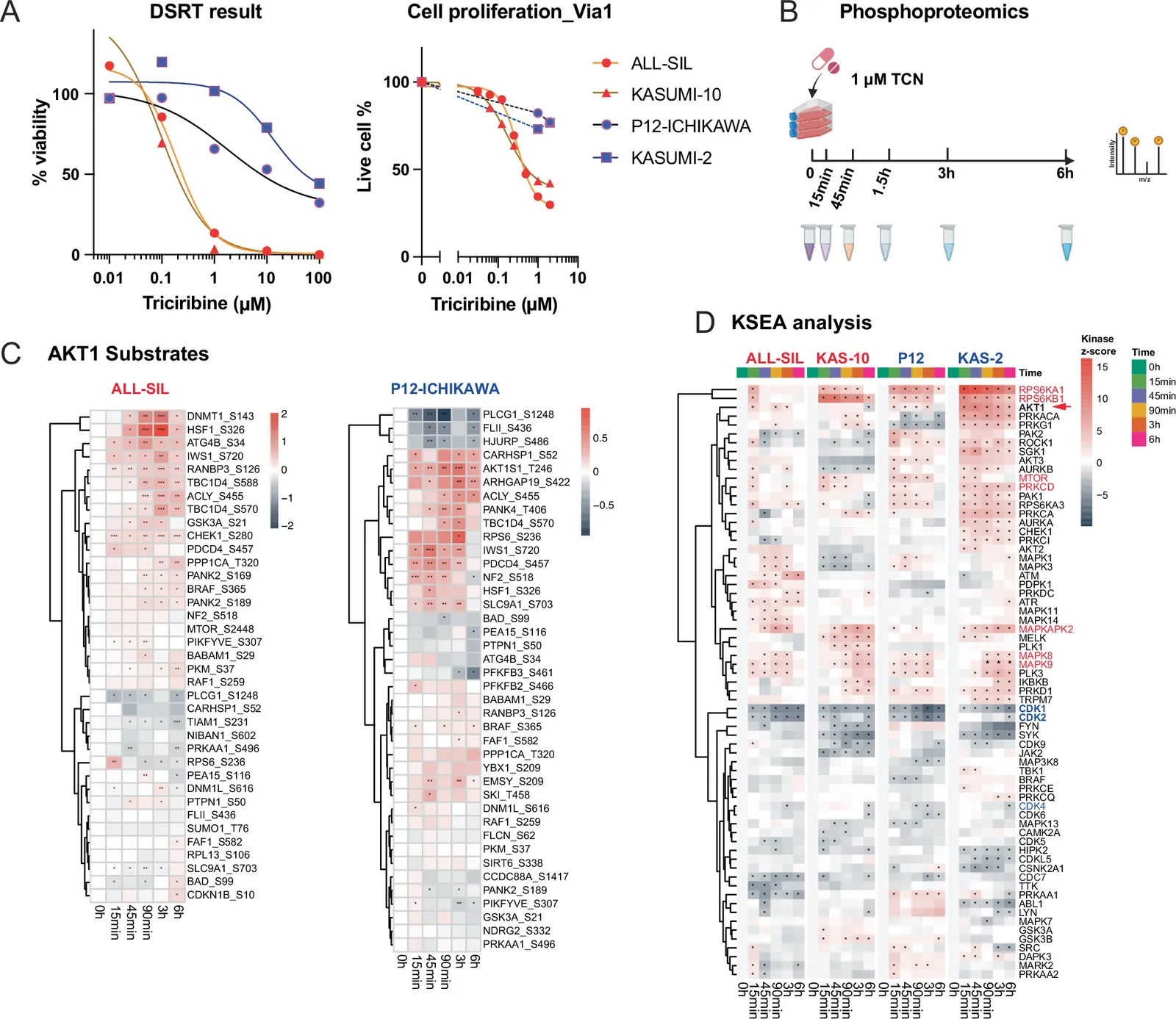
Phosphoproteomic analysis of signaling responses following triciribine treatment. **A** Drug sensitivity testing (DSRT) and in-house cell proliferation assays comparing TCN sensitivity in four ALL cell lines (ALL-SIL, P12-ICHIKAWA, KASUMI-10, and KASUMI-2). Cells were treated with increasing concentrations of TCN for 72 h, and cell number and viability were measured using CellTiter-Glo or Via1 cassette-NucleoCounter, respectively. For P12-ICHIKAWA and KASUMI-2, the Via1 validation assay focused on higher TCN concentrations, and low-concentration points were therefore not measured. **B** Experimental design and workflow for time-course phosphoproteomic analysis. Four ALL cell lines (ALL-SIL, P12-ICHIKAWA, KASUMI-10, and KASUMI-2) were treated with 1 μM TCN for the indicated time points (*n* = 3 replicates per time point) prior to phosphopeptide enrichment and LC-MS/MS analysis. **C** Phosphorylation changes of known Akt1 substrates across time points relative to 0 h in ALL-SIL and P12-ICHIKAWA cells. Statistical comparisons were performed using a two-tailed unpaired Welch’s *t*-test. Statistical significance: * *p* < 0.05; ** *p* < 0.01, *** *p* < 0.001. D Heatmap of kinase activity scores derived from KSEA across all time points relative to 0 h in four ALL cell lines (ALL-SIL, P12-ICHIKAWA [P12], KASUMI-10 [KAS-10], and KASUMI-2 [KAS-2]). The *p*-value from KSEA analysis is determined by assessing the one-tailed probability of having a more extreme score than the one measured, followed by a Benjamini–Hochberg FDR correction for multiple hypothesis testing. Kinases with KSEA *p* < 0.05 are marked with an asterisk.

To systematically characterize kinase signaling responses to TCN, we performed time-course phosphoproteomic analyses in four ALL cell lines representing different lineages, immunophenotypes, and sensitivities: ALL-SIL (T-ALL, ABL1 rearranged, sDSS = 19.5), P12-ICHIKAWA (T-ALL, sDSS = 2.8), KASUMI-10 (BCP-ALL, KMT2A rearranged, sDSS = 24.2), and KASUMI-2 (BCP-ALL, TCF3 rearranged, sDSS = −4.7) (Fig. 2A). ALL-SIL and KASUMI-10 were classified as TCN-sensitive, whereas P12-ICHIKAWA and KASUMI-2 showed intermediate or resistant TCN responses. The high-throughput drug screening results were consistent with independent methods of viability measurement, despite higher apparent IC50 values and methodological differences between assays (Fig. 2A).

Based on IC50 values below or near 1 µM in sensitive lines, we selected 1 µM TCN for phosphoproteomic analyses. Samples were collected at baseline and at 15 min, 45 min, 1.5 h, 3 h, and 6 h after treatment, with three biological replicates per time point (Fig. 2B). Principal component analysis demonstrated clear temporal separation following TCN exposure, with tight clustering of replicates, indicating robust phosphoproteomic measurements (Supplementary Fig. 2A). In contrast, global protein abundance showed minimal changes during the early time points (Supplementary Fig. 2B), supporting independent analysis of phosphoproteomic data.

Analysis of known Akt substrates, curated from PhosphoSitePlus, revealed that phosphorylation of the majority of Akt substrates increased following TCN treatment across all four cell lines, despite variability in individual substrate dynamics (Fig. 2C and Supplementary Fig. 2C). Phosphorylation levels typically peaked between 45 min and 3 h, broadly consistent with the transient early Akt phosphorylation observed by immunoblotting, although the precise timing differed between assays (Fig. 1D).

Kinase-substrate enrichment analysis (KSEA) was performed using baseline samples as controls^36^. Across all 4 cell lines, cell-cycle-associated kinases CDK1, CDK2, and CDK4 were consistently inhibited following TCN treatment (Fig. 2D). In contrast, kinases within the mTOR pathway (mTOR, RPS6KB1) and MAPK signaling pathways (MAPKAPK2, MAPK8/9, RPS6KA1) were consistently activated. Akt1 activity was transiently increased in all four cell lines at early time points, with reduced activity observed at later time points in selected models.

Comparison of KSEA profiles across the four cell lines revealed predominantly shared early kinase responses to TCN, with relatively few changes consistently distinguishing sensitive from less-sensitive models (Fig. 2D and Supplementary Fig. 2D). Among these, PAK1 was activated in both less-sensitive cell lines but showed no significant activation in the sensitive models. PRKAA1/AMPKα1 also showed activation at one or more early time points in both less-sensitive models, whereas inhibition or no significant change was observed in the sensitive cell lines. ROCK1 similarly showed activation in both less-sensitive models, whereas inhibition or no significant change was observed in the sensitive models, with this contrast becoming most apparent from 45 min onward. These observations identify PAK1, PRKAA1, and ROCK1 as candidate differential signaling responses observed consistently in the two less-sensitive models; however, their temporal variability, the limited number of models, and the absence of functional validation preclude assignment of a direct role in TCN resistance.

Overall, these results demonstrate that TCN induces reproducible and predominantly shared early phosphoproteomic responses across ALL cell lines, characterized by CDK inhibition and activation of mTOR- and MAPK-associated signaling, while a limited number of kinase responses may differ according to TCN sensitivity. Together with the predominantly increased phosphorylation of Akt substrates, these kinase activity profiles indicate that early TCN exposure does not induce consistent suppression of Akt pathway signaling across the four ALL models.

### PISA profiling identifies candidate triciribine-interacting proteins across the proteome

**Fig. 3:**
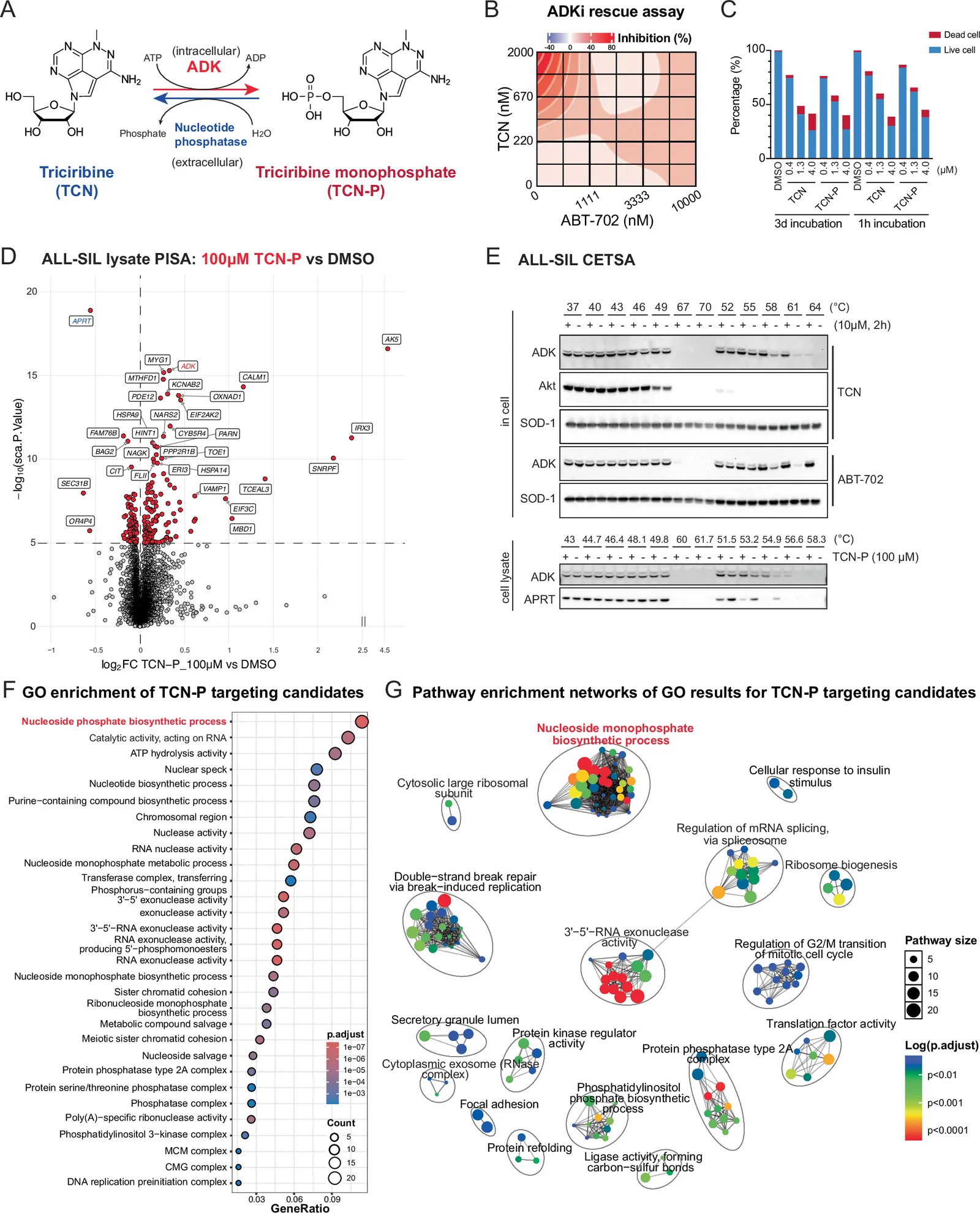
Identification of candidate triciribine-interacting proteins by PISA profiling. **A** Chemical structures of TCN and TCN-P, and schematic representation of their interconversion by ADK and nucleotide phosphatases. ATP adenosine triphosphate; ADP adenosine diphosphate. **B** Cell viability and cell number measurements in ALL-SIL cells treated with varying concentrations of TCN and the ADK inhibitor (ADKi) ABT-702, alone or in combination, for 3 days. Live cell numbers were quantified using the Via1 cassette-NucleoCounter and used as input for SynergyFinder to evaluate the rescue effect of ABT-702 on TCN-induced cytotoxicity. **C** Cell viability and cell number were measured on day 3 using the Via1 cassette-NucleoCounter in ALL-SIL cells following short-term (1 h) or continuous (3-day) exposure to different concentrations of TCN or TCN-P; one measurement was obtained per concentration. The percentages of live and dead cells were normalized to the corresponding DMSO control within each treatment group. **D** Volcano plot of PISA results showing changes in protein solubility following treatment with 100 μM TCN-P compared with DMSO control. Statistical analysis was performed using DEqMS, and DEqMS-derived *p*-values (sca.P.Value) are shown on the *y*-axis, and log_2_FC (fold change) on the *x*-axis. **E** Cellular and lysate-based CETSA immunoblots demonstrating thermal stabilization of ADK, and in lysate CETSA, APRT following treatment with TCN or TCN-P. The highest-temperature samples were loaded in the central wells of the 26-well gel to minimize the impact of poorer band quality associated with these wells; therefore, temperature points are not displayed in sequential order. **F** Gene Ontology (GO) enrichment analysis of candidate TCN-P interacting proteins identified by PISA. Dot plots representing the top enriched terms for each GO domain, with dot size indicating the number of genes involved in a specific pathway or process and color representing statistical significance. **G** Pathway enrichment network visualization of GO terms enriched among candidate TCN-P interacting proteins.

Given the limited early inhibition of Akt signaling, we next sought to identify candidate direct protein interactions of TCN using PISA profiling^30^. Prior studies have shown that TCN is rapidly transported into cells and phosphorylated by ADK to generate the predominant intracellular species TCN-P, resulting in substantial intracellular accumulation^13^ (Fig. 3A).

We confirmed that TCN-P is the dominant intracellular form of TCN in ALL cells with LC–MS/MS. Pharmacologic inhibition of ADK using ABT-702 or 5-Iodotubercidin^11^ rescued TCN-induced cytotoxicity in ALL-SIL cells (Fig. 3B and Supplementary Fig. 3A), confirming the requirement of ADK-mediated phosphorylation. Short-term exposure to TCN or TCN-P followed by drug washout was sufficient to induce cytotoxicity at day 3 comparable to that observed with continuous treatment, and similar inhibitory effects were observed for both compounds^11^ (Fig. 3C). These findings indicate rapid cellular uptake and conversion of TCN to TCN-P and are consistent with previous observations^13^.

Based on published intracellular concentration estimates^13^, PISA profiling was performed using ALL-SIL lysate treated with 100 µM TCN-P to approximate intracellular exposure corresponding to 1 µM extracellular TCN. For comparison, 100 µM TCN and 1 µM TCN-P were included to represent the prodrug and low-dose conditions, respectively. Four biological replicates were analyzed for each condition (Supplementary Fig. 3B).

Relative to vehicle control, 100 µM TCN-P yielded the largest number of candidate interacting proteins (Fig. 3D and Supplementary Fig. 3C, D). ADK emerged as the top hit for both TCN and TCN-P, consistent with in-cell and lysate CETSA (cellular thermal shift assay) validation (Fig. 3E and Supplementary Fig. 3E). In contrast, no significant thermal stabilization of Akt was observed (Fig. 3E and Supplementary Fig. 3F), whereas multiple components of the Akt regulator PI3K and PP2A complexes showed altered stability following TCN-P treatment (Supplementary Fig. 3G, H). These observations may provide context for the modest correlations observed between TCN and selected PI3K or PI3K/mTOR inhibitors (Fig. 1C), as well as the context-dependent effects on Akt signaling^17^.

To define high-confidence candidate interactions, we applied a stringent DEqMS scaled *p*-value threshold (sca.P.Value < 10⁻⁵), yielding 199 candidate TCN-P-interacting proteins with at least one identified peptide (Fig. 3D). Gene ontology analysis revealed enrichment across diverse biological processes, with nucleoside phosphate biosynthetic processes representing the most significant cluster, along with DNA replication, RNA-associated processes, protein translation, protein folding, and cell cycle-related processes (Fig. 3F, G).

### TCN-P engages multiple metabolic and biosynthetic pathways and induces broad cellular stress responses in ALL-SIL cells

**Fig. 4:**
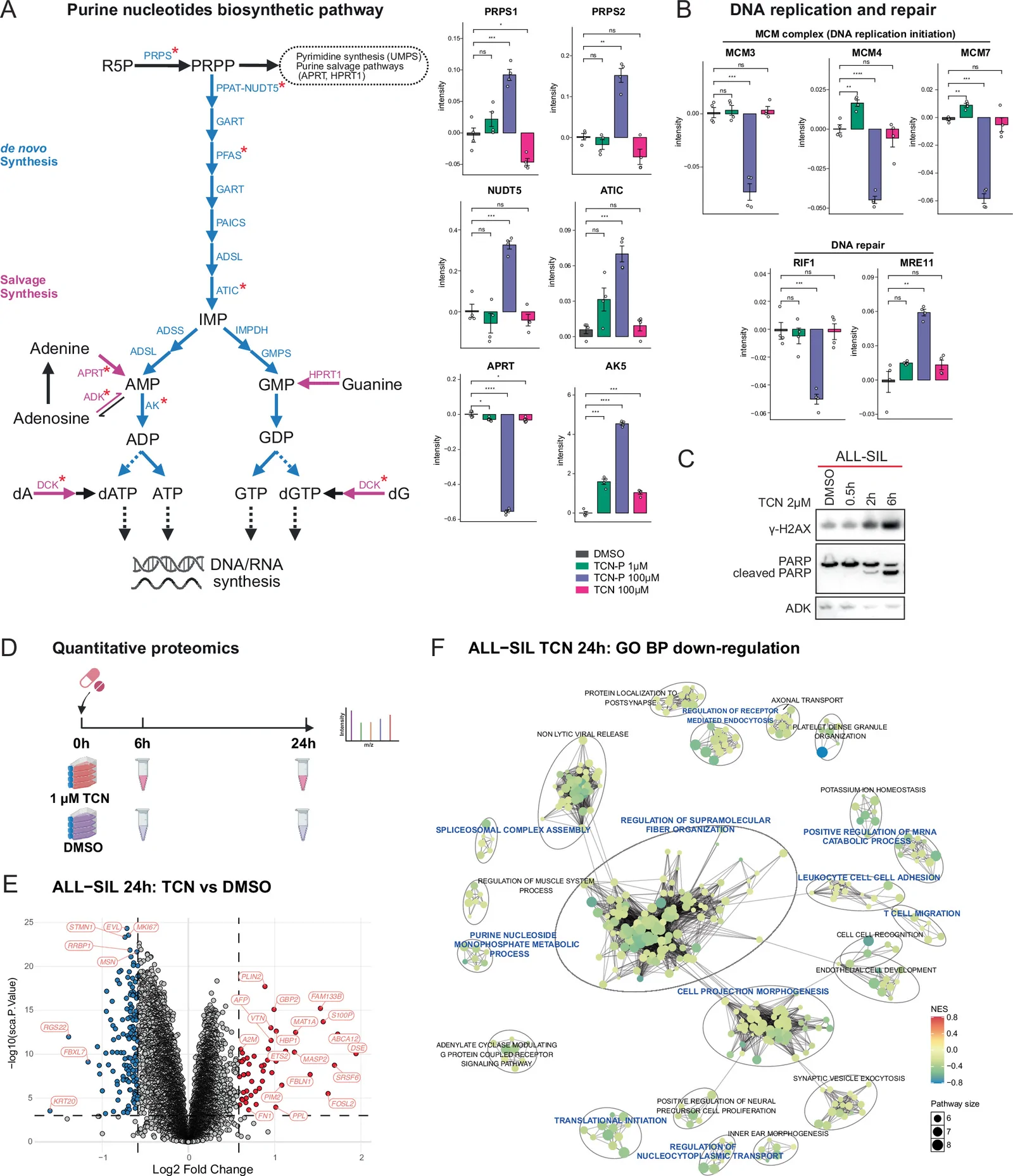
Proteomic characterization of cellular responses to triciribine in ALL-SIL cells. **A** Schematic overview of de novo and salvage purine nucleotide biosynthesis pathways. Enzymes identified as candidate TCN-P interacting proteins are indicated with asterisks (*). Bar plots show candidate TCN-P interacting proteins involved in purine nucleotide biosynthesis pathways (*n* = 4 replicates per condition). Statistical comparisons were performed using a two-tailed unpaired Welch’s *t*-test. Statistical significance is indicated as * *p* < 0.05; ** *p* < 0.01, *** *p* < 0.001. **B** Bar plots showing candidate TCN-P interacting proteins involved in DNA replication and repair. Colors representing each condition are shown in (**A**). **C** Immunoblot analysis of γ-H2AX and PARP in ALL-SIL cells following TCN treatment. **D** Experimental design and workflow for quantitative proteomic analysis. Four ALL cell lines were treated with DMSO or 1 μM TCN for 6 or 24 h (*n* = 4 replicates per time point) prior to LC–MS/MS analysis. **E** Volcano plot of differential protein abundance in ALL-SIL cells treated with TCN for 24 h compared with DMSO. Statistical analysis was performed using DEqMS, and DEqMS-derived *p*-values (sca.P.Value) are shown on the *y*-axis, and Log_2_ Fold Change on the *x*-axis. **F** Visualization of significantly downregulated biological process networks identified by ssGSEA in TCN-treated ALL-SIL cells.

Previously reported reductions in intracellular ATP and GTP pools and inhibition of nucleic acid synthesis following TCN exposure^13^ are in line with our PISA findings, which identified stability shifts in multiple proteins involved in de novo and salvage purine biosynthesis or its regulation, including ADK, APRT, AK5, PRPS, NUDT5, ATIC, and MTHFD1 (Fig. 4A, Fig. 3D, and Supplementary Fig. 3E). Although AK5 showed one of the strongest changes in the PISA analysis, it was not prioritized as an individual candidate because its quantification was supported by only one unique peptide. Nevertheless, its role in nucleotide homeostasis is consistent with the broader enrichment of purine nucleotide metabolism observed in the PISA dataset.

In addition to purine metabolism, candidate TCN-P interactions were identified among proteins involved in DNA replication and repair, including components of the MCM complex, RIF1, and MRE11^37,38^ (Fig. 4B). While TCN-P is not incorporated into nucleic acids, these interactions suggest that its cellular effects may extend beyond direct metabolic inhibition to broader biosynthetic and stress-related processes. Consistent with this notion, TCN treatment induced DNA damage at early time points, as evidenced by γ-H2AX induction^39^, and subsequent apoptotic signaling indicated by PARP cleavage, preceding detectable changes in cell proliferation or viability (Fig. 4C).

To assess the downstream consequences of this broad interaction landscape, we performed time-course quantitative proteomics in ALL-SIL cells at 6 h and 24 h following TCN treatment (Fig. 4D). Although relatively few proteins exhibited large changes in abundance at these early time points (Fig. 4E), pathway-level analyses revealed coordinated alterations across multiple biological processes (Supplementary Fig. 4A). Single-sample GSEA was consistent with suppression of purine biosynthesis and protein translation, alongside induction of DNA damage response pathways, proteotoxic stress responses, and alterations in cytoskeletal organization and intracellular transport (Fig. 4F and Supplementary Fig. 4B).

Collectively, these findings indicate that TCN-P engages a diverse set of cellular proteins and pathways, resulting in broad metabolic and biosynthetic stress rather than disruption of a single dominant target or process. In ALL-SIL cells, TCN exposure is therefore associated with a pleiotropic cellular response consistent with antimetabolite activity.

### ADK abundance correlates with triciribine sensitivity, and comparative proteomics across ALL models highlights sensitivity-associated response programs

**Fig. 5:**
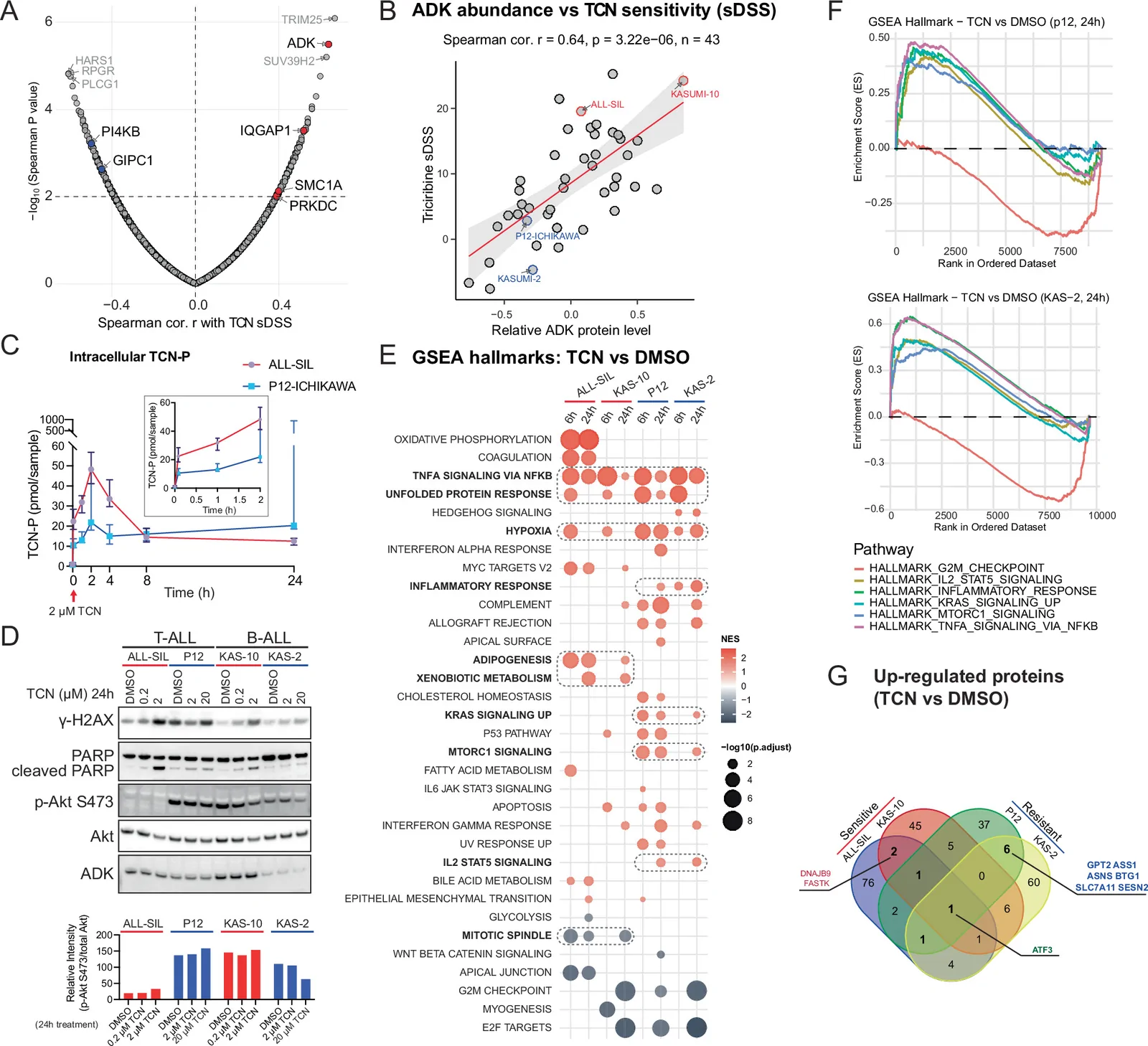
Association of ADK abundance with triciribine sensitivity and comparative proteomic responses across ALL models. **A** Spearman correlation analysis between baseline protein abundance and TCN sensitivity (sDSS) across 43 ALL cell lines. Significant candidate TCN-P interacting proteins are highlighted with black labels. Data were obtained from the FORALL database. **B** Correlation between TCN sensitivity (sDSS) on the *y*-axis and ADK protein abundance on the *x*-axis in 43 ALL cell lines. Data were obtained from the FORALL database. **C** Time-course quantification of intracellular TCN-P concentrations in ALL-SIL and P12-ICHIKAWA cells following treatment with 2 μM TCN. Six replicates were analyzed at each time point. One measurement from the P12-ICHIKAWA 1 h group, one from the P12-ICHIKAWA 2 h group, and one from the ALL-SIL 24 h group were identified as outliers using the IQR method (1.5× IQR criterion) and excluded from the plotted dataset. Bars represent the median and interquartile range (25th–75th percentile). **D** Immunoblot analysis of γ-H2AX, PARP, phosphorylated Akt (Ser473), and total Akt in ALL cell lines following treatment with increasing concentrations of TCN. The lower panel shows intensity quantification of the p-Akt (Ser473)/total Akt ratio from the corresponding immunoblots. **E** Hallmark gene set enrichment analysis (GSEA) comparing total proteome changes between 1 μM TCN- and DMSO-treated samples at 6 and 24 h across four ALL cell lines (ALL-SIL, P12-ICHIKAWA [P12], KASUMI-10 [KAS-10], and KASUMI-2 [KAS-2]). **F** Representative GSEA enrichment plots of pathways consistently upregulated in less-sensitive cell lines P12-ICHIKAWA and KASUMI-2 following 1 μM TCN treatment compared to DMSO at 24 h. **G** Venn diagram showing the overlap of proteins significantly upregulated following TCN treatment at both 6 and 24 h across four ALL cell lines (ALL-SIL, P12-ICHIKAWA [P12], KASUMI-10 [KAS-10], and KASUMI-2 [KAS-2]).

Given the broad interaction profile of TCN-P, we next examined whether the abundance of candidate interacting proteins correlated with TCN sensitivity across ALL models. Pairwise correlation analysis of baseline protein levels and TCN drug sensitivity across 43 ALL cell lines identified ADK as the interacting protein most strongly associated with TCN response (Fig. 5A, B). This association was recapitulated in the four cell lines selected for proteomic analyses, consistent with prior transcript-level observations (Fig. 5B and Supplementary Fig. 5A). In addition to ADK, TRIM25 abundance correlated with TCN sensitivity in the FORALL dataset. However, this association was not reproduced in the PRISM dataset, suggesting that it may reflect an ALL-specific context rather than a broadly applicable biomarker.

To determine whether ADK abundance influences intracellular TCN-P dynamics, we performed time-course LC–MS/MS quantification of TCN and TCN-P in ALL-SIL (ADK-high, sensitive) and P12-ICHIKAWA (ADK-low, less-sensitive) cells following exposure to TCN. In both cell lines, intracellular TCN-P levels increased rapidly, peaking within approximately 2 h, and subsequently declined to a stable plateau, consistent with (partial) export of TCN-P into the extracellular compartment^40^ (Fig. 5C and Supplementary Fig. 5B). Notably, the maximal TCN-P concentration achieved differed substantially between the two models and was substantially higher in the ADK-high ALL-SIL model, whereas steady-state TCN-P levels were comparable. The intracellular TCN-P concentration reached in ALL-SIL cells within 1 h exceeded the maximal level observed in P12-ICHIKAWA cells, supporting the notion that short-term exposure is sufficient to induce cytotoxicity in ADK-high models (Fig. 5C and Fig. 3C). In contrast, intracellular TCN levels remained low and comparable between cell lines throughout the time course (Supplementary Fig. 5B). Although these measurements were performed in two representative models with contrasting ADK abundance and TCN sensitivity, validation across a larger panel will be required to establish the general relationship between ADK abundance and intracellular TCN-P pharmacokinetics in ALL.

Increasing extracellular TCN concentrations in ADK-low cells failed to further enhance DNA damage or apoptotic responses (Fig. 5D), indicating that ADK abundance limits maximal intracellular formation of TCN-P and, consequently, the magnitude of downstream cellular stress.

Although ADK-dependent activation is required for TCN cytotoxicity, differences in ADK level alone did not fully explain the observed variation in drug sensitivity across ALL models. We therefore compared early proteomic response programs across cell lines with differing TCN sensitivities. Using the same quantitative proteomics workflow applied to ALL-SIL, we analyzed TCN-induced proteomic changes in P12-ICHIKAWA, KASUMI-10, and KASUMI-2 cells (Fig. 4D). Hallmark pathway analysis revealed both shared and divergent response patterns (Fig. 5E). Across all models, TCN treatment was associated with enrichment of unfolded protein response, hypoxia, and NF-κB signaling pathways, consistent with generalized cellular stress. In contrast, suppression of mitotic and proliferative programs was more pronounced in sensitive models, whereas less-sensitive models exhibited stronger induction of adaptive stress and survival-associated pathways, including mTORC1, KRAS-related, and inflammatory signaling (Fig. 5F). Several stress-associated proteins, including GPT2, SLC7A11, ASNS, and SESN2^41,42^, showed greater induction in less-sensitive cells following TCN treatment (Fig. 5G and Supplementary Fig. 5C–E).

Together, these results suggest that ADK abundance governs the extent of intracellular TCN-P accumulation and thereby influences the magnitude of TCN-induced cellular stress. Downstream proteomic responses vary across ALL models and reflect differential engagement of adaptive pathways rather than activation of a single resistance mechanism.

### Associations between ADK level and triciribine response in clinical samples

**Fig. 6:**
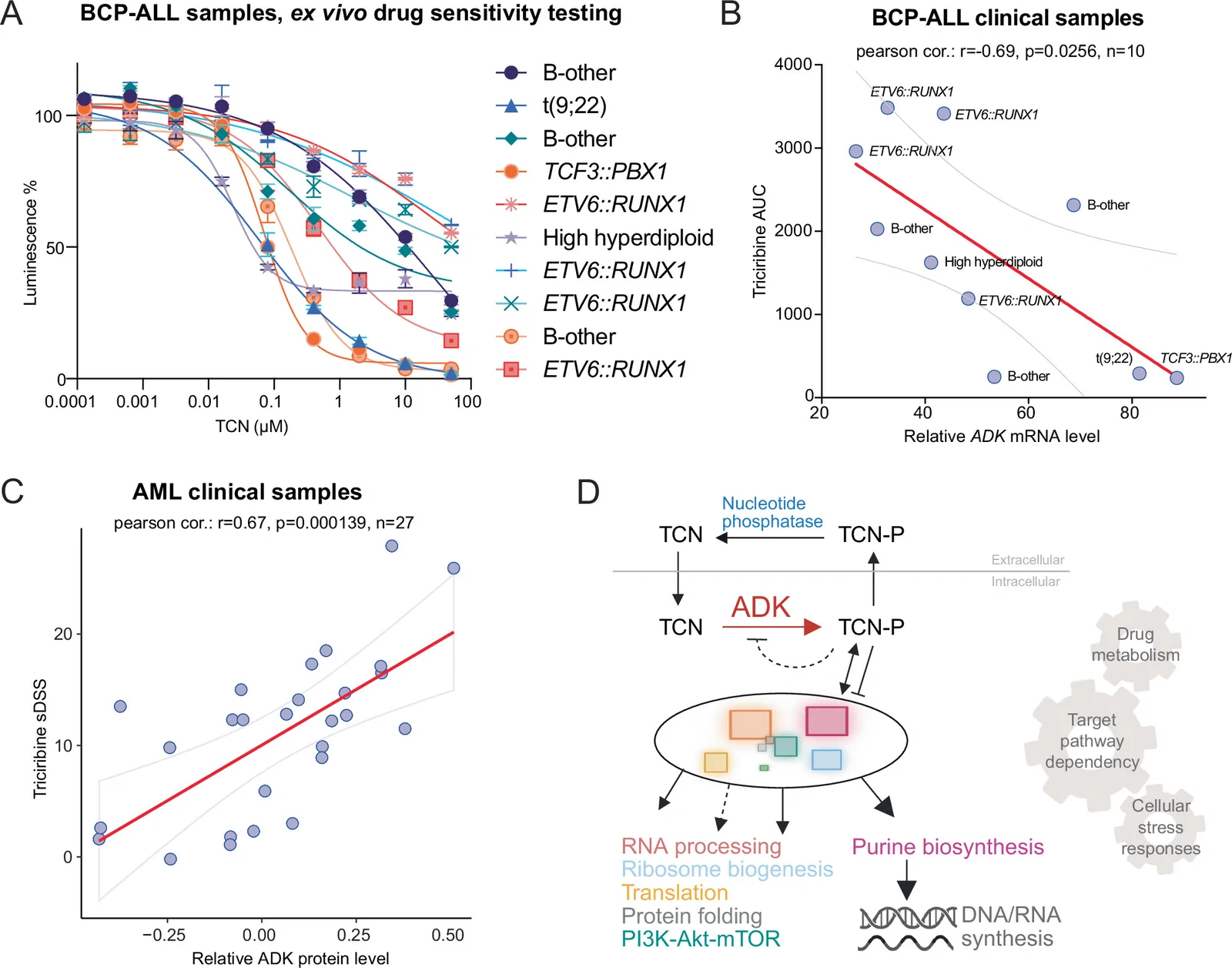
Ex vivo triciribine sensitivity and association with ADK level in clinical samples. **A** Ex vivo drug sensitivity testing of TCN in primary BCP-ALL samples (*n* = 10). Cells were incubated with TCN for 72 h, followed by measurement of ATP content using the CellTiter-Glo assay. Results are presented as mean ± SD of three replicates per concentration. **B** Correlation between TCN sensitivity (AUC, area under the curve) and *ADK* mRNA level in BCP-ALL samples. Lower AUC values indicate increased TCN sensitivity. The Pearson correlation test was used. **C** Correlation between TCN sensitivity (sDSS) and ADK protein abundance in AML samples. **D** Schematic working model summarizing proposed cellular processes associated with TCN activity in ALL cells.

To explore clinical relevance, we assessed TCN sensitivity in primary BCP-ALL samples ex vivo. TCN at 1 µM achieved approximately 50% growth inhibition in five out of ten samples (Fig. 6A). Higher *ADK* mRNA levels were significantly associated with greater TCN sensitivity (lower AUC) (Fig. 6B). Consistent with the association observed in ALL samples, ex vivo testing in acute myeloid leukemia (AML) cohorts also showed increased TCN sensitivity in samples with higher ADK levels^43–45^ (Fig. 6C). In an AML cohort of 488 patient samples from KM plotter platform^46^, high ADK expression was associated with inferior prognosis in chemotherapy-treated patients, but not in untreated patients (Supplementary Fig. 6A, B), identifying ADK-high AML as a potential context of interest for future evaluation of TCN.

Together, these findings suggest that ADK level contributes to cellular responses to TCN (Fig. 6D).

## Discussion

In this study, we applied integrated proteomic approaches to characterize the cellular responses and candidate targets associated with TCN treatment in ALL. Across multiple ALL models, TCN exhibited potent antileukemic activity and a response profile distinct from canonical Akt inhibitors. Rather than inducing immediate suppression of Akt signaling, phosphoproteomic analyses revealed early inhibition of cyclin-dependent kinases accompanied by activation of stress-associated signaling pathways, including mTOR and MAPK, with evidence of variable Akt modulation at later time points. Together, these findings suggest that TCN is unlikely to function predominantly through immediate and direct suppression of Akt signaling in ALL, but instead elicits a broader cellular stress response consistent with indirect and multi-layered mechanisms of action. Later effects on Akt pathway activity may emerge downstream of cellular stress.

Using PISA profiling, we identified a diverse landscape of candidate protein interactions for the active metabolite TCN-P. These interactions were distributed across multiple metabolic and biosynthetic pathways, including nucleotide metabolism, DNA replication, protein synthesis, and RNA-associated processes. Rather than pointing to a single dominant downstream target, this interaction profile supports a model in which TCN-P perturbs multiple cellular systems simultaneously, consistent with the behavior of endogenous metabolites, which frequently interact with multiple proteins across functionally distinct pathways^47^. Consistent with this interpretation, quantitative proteomics revealed coordinated pathway-level changes following TCN exposure, including suppression of purine biosynthesis and translational programs alongside activation of DNA damage responses and proteotoxic stress pathways. These findings collectively characterize TCN as a pleiotropic antimetabolite whose cytotoxic effects arise from broad biosynthetic disruption rather than selective inhibition of an individual enzyme or signaling node.

A key finding of this study is the identification of ADK as a key determinant of TCN sensitivity across ALL models. ADK catalyzes the conversion of TCN to TCN-P, the predominant intracellular and biologically active species, and ADK abundance showed the strongest correlation with drug sensitivity among candidate interacting proteins. Kinetic analyses further demonstrated that ADK levels may govern the maximal intracellular accumulation of TCN-P, thereby shaping the magnitude of downstream cellular stress. Importantly, increasing extracellular TCN concentrations failed to overcome low ADK levels, indicating that intracellular activation rather than drug availability limits cytotoxic efficacy. These observations position ADK as a critical gatekeeper of TCN activity and a rational candidate biomarker for patient stratification.

Although ADK abundance strongly influences TCN sensitivity, our data also suggest that downstream cellular context contributes to differential responses. Comparative proteomic analyses across ALL models revealed shared stress-associated responses following TCN exposure, alongside variability in adaptive signaling programs. Less-sensitive models preferentially activated pathways associated with metabolic adaptation and survival, including mTORC1, KRAS-related signaling, and inflammatory responses, whereas suppression of proliferative programs was more pronounced in sensitive cells. These findings support a model in which TCN sensitivity reflects both efficient intracellular activation and the capacity, or failure, of leukemic cells to engage compensatory stress responses, rather than dependence on a single resistance mechanism.

Our findings also have implications for the clinical development of TCN. Although the present study primarily focuses on ALL, the association between ADK levels and TCN sensitivity appears consistent across hematologic malignancies. Previous clinical studies have reported limited objective responses despite evidence of biological activity and acceptable toxicity profiles. Notably, intracellular accumulation of TCN-P in patient leukemic blasts has been substantially lower than that observed in cell line models^24^, raising the possibility that insufficient intracellular activation, rather than intrinsic insensitivity of downstream targets, may have constrained therapeutic efficacy. Consistent with this notion, our PISA analyses demonstrated dose-dependent TCN-P-associated changes in protein solubility profiles across 0 μM (DMSO), 1 μM, and 100 μM TCN-P conditions, suggesting that suboptimal intracellular concentrations may fail to induce the broad biosynthetic stress required for effective cytotoxicity. These observations underscore the importance of considering intracellular drug metabolism and activation, in addition to target engagement, when evaluating antimetabolite therapies. Rather than supporting direct replacement of established antimetabolites used in ALL treatment, the distinct metabolism and ADK-dependent activation of TCN provide a rationale for its further evaluation in biomarker-selected patients, either as a single agent or in rational combinations with existing ALL therapies. Future studies directly comparing TCN with clinically used antimetabolites and evaluating rational combinations will be required to determine whether its distinct mechanism and ADK-based patient stratification can provide additional therapeutic benefit.

Several limitations of this study should be acknowledged. First, while proteomic approaches provide an unbiased and systems-level view of drug responses, they can not distinguish primary from secondary effects with certainty. Some observed proteomic changes may therefore reflect downstream consequences of metabolic disruption rather than direct TCN-P interactions. Second, the cell lysate-based PISA approach may underrepresent membrane-associated proteins and transporters, potentially missing factors involved in TCN/TCN-P uptake^12^, export^40^, degradation^48^, or dephosphorylation that could influence intracellular drug exposure. More generally, PISA is limited to proteins that are detected and quantified by mass spectrometry and to interactions that induce measurable solubility changes; therefore, the absence of a protein from the PISA dataset does not exclude its potential involvement in TCN-P activity. For example, the proposed TCN-P efflux transporter ABCC1/MRP1 did not show a significant PISA solubility change, although it may contribute to TCN-P efflux^49^, and its protein abundance was modestly negatively correlated with TCN sensitivity in the FORALL dataset. Third, although CETSA and quantitative proteomics provided orthogonal and functional support for selected candidate interactions, PISA-associated solubility changes do not by themselves establish direct binding, and comprehensive validation of individual targets through genetic or biochemical approaches was beyond the scope of this study. Finally, in vivo pharmacokinetic and pharmacodynamic relationships remain to be fully defined and will be essential for translating these findings into clinical strategies.

In summary, our study provides a comprehensive proteomic characterization of TCN activity in ALL and supports a model in which TCN functions as a pleiotropic antimetabolite whose activity depends on intracellular activation through ADK and is further shaped by downstream cellular context. By defining the interaction landscape and downstream cellular responses associated with TCN-P, this work establishes a framework for biomarker-guided evaluation of TCN and highlights the importance of drug metabolism and biosynthetic stress in shaping antileukemic responses.

## Methods

### Cell lines and cell culture

ALL-SIL, P12-ICHIKAWA, and KASUMI-2 cells were maintained in RPMI 1640 medium containing 2 mM stable glutamine (L-Ala-L-Gln dipeptide; Sigma-Aldrich), supplemented with 10% fetal bovine serum (FBS; Sigma-Aldrich), 20 mM HEPES (Gibco), 1 mM sodium pyruvate (Sigma-Aldrich), 1× MEM nonessential amino acids (Sigma-Aldrich), and 1× penicillin-streptomycin (Sigma-Aldrich), and cultured at 37 °C in a humidified atmosphere with 5% CO₂. KASUMI-10 cells were maintained in Iscove’s Modified Dulbecco’s Medium (IMDM; Sigma-Aldrich) supplemented with 20% FBS and 1× penicillin–streptomycin. Cell line origin and authentication information have been reported previously^32^. All cell lines were routinely tested for mycoplasma contamination using the MycoAlert Mycoplasma Detection Kit (Lonza) and authenticated by short tandem repeat (STR) profiling (Eurofins Genomics). Total and viable cell numbers were routinely assessed using the Via1 cassette, containing the fluorophores acridine orange (AO) and 4′,6-diamidino-2-phenylindole (DAPI), in combination with the NucleoCounter system (ChemoMetec) for cell viability monitoring and proliferation assays.

### Clinical samples and drug sensitivity assay

Primary ALL samples were obtained with informed consent under protocols approved by the Regional Ethical Review Board in Stockholm (2015/1998-3; 2003/02-445; 2018/38-32). All experiments involving patient material were performed ex vivo, and no clinical interventions were conducted. For TCN sensitivity testing, cells were resuspended in Hematopoietic Progenitor Expansion Medium DXF supplemented with Cytokine Mix E (Merck; C-28021, C-39891) and seeded at 1 × 10⁶ cells/mL in 96-well flat-bottom white plates (Thermo Fisher Scientific; Cat. No. 136101). DMSO levels were normalized across the plate with a maximum final concentration of 0.1%. TCN was serially diluted to final concentrations ranging from 0.0001 to 50 µM in a 96-well plate format. Cells were incubated with TCN for 72 h, after which cell viability was measured using CellTiter-Glo 2.0 ATP reagent (Promega; G9242) on a Victor 3 V plate reader. Relative cell viability values were subsequently curve-fitted using a nonlinear regression model (four-parameter sigmoidal model) in GraphPad Prism 11 to determine area under the curve (AUC) values where applicable.

Additional analyses were performed using an AML proteomics and drug sensitivity dataset comprising 27 patient samples, with ethical approvals issued by the Swedish Ethical Review Authority (2017/285-31/2 and 2025-08490-01). The complete dataset will be made publicly available upon publication of the original study, including the associated proteomics data in PRIDE (PXD061758). Portions of the dataset and associated analyses have been reported previously^45^.

Clinical trial number: not applicable.

### Reagents and antibodies

TCN (A8541), ABT-702 (C5282), and 5-Iodotubercidin (A3125) were purchased from ApexBio Technology. TCN-P (37985) was obtained from Cayman Chemical. Primary antibodies included ADK (AM8619b-ev; Nordic Biosite), APRT (LS‑C748961; LSBio), SOD1 (sc-17767; Santa Cruz Biotechnology), PARP (9532), Akt (4691), Phospho-Histone H2A.X (Ser139; 2577), and phospho-Akt (Ser473; 4060) (Cell Signaling Technology). LI-COR IRDye secondary antibodies were used for detection.

### In-cell CETSA

In-cell CETSA was performed as described previously^32,50^. ALL-SIL cells (1.0 × 10⁶ cells/mL) were treated with TCN (10 μM), ABT-702 (10 μM), or DMSO for 2 h at 37 °C. Cells were aliquoted and subjected to a thermal challenge across a temperature range of 37.0–70.0 °C (12 points with 3.0 °C increments) for 3 min, followed by cooling at room temperature for 3 min and snap-freezing. Cell lysates were prepared by four freeze-thaw cycles. Aggregated proteins were removed by centrifugation at 20,000×*g* for 30 min at 4 °C, and the soluble protein fraction was collected for western blot analysis.

### Cell lysate CETSA

ALL-SIL cell lysates were prepared by freeze-thaw lysis in 100 mM HEPES containing 20 mM MgCl₂. Cleared lysates were incubated with 100 μM TCN-P or DMSO for 5 min at room temperature prior to thermal challenge. Samples were aliquoted and heated across the same temperature range used for the PISA assay (43.0–61.7 °C; 12 temperature points with 1.7 °C increments), followed by cooling at room temperature for 3 min. Aggregated proteins were removed by centrifugation at 20,000×*g* for 30 min at 4 °C, and the soluble protein fraction was collected for western blot analysis.

### Quantitative proteomics

Quantitative proteomics was performed as previously described^32^, with modifications described below. Unless otherwise indicated, cell centrifugation steps were performed at 500 × *g* for 3 min. Cells were adjusted to 1 × 10⁶ cells/mL and treated with 1 μM TCN (prepared from a 1 mM stock in DMSO) or the corresponding DMSO vehicle control (*n* = 4 replicates per condition). For each replicate, 5 mL of cell suspension was harvested after 6 or 24 h of treatment. Cells were collected by centrifugation and washed twice with calcium-, magnesium-, and phenol red-free Hank’s Balanced Salt Solution (HBSS; Gibco). Cell pellets were immediately lysed in 100 μL lysis buffer containing 4% SDS, 25 mM HEPES (pH 7.6), and 1 mM DTT. Lysates were heated at 95 °C for 5 min and stored at −80 °C until further processing.

After all samples had been collected, lysates were thawed and sonicated (Bandelin). Insoluble material was removed by centrifugation at 14,000 × *g* for 15 min, and total protein concentration was determined using the Bio-Rad DC Protein Assay. Peptides were prepared using a modified single-pot, solid-phase-enhanced sample preparation (SP3) protocol^51^. Briefly, 300 μg protein per sample was alkylated with chloroacetamide at a final concentration of 4 mM for 10 min. Samples were combined with SP3 beads, and acetonitrile (ACN) was added to a final concentration >70%. Following incubation for 30 min with gentle rotation, beads were washed with 70% ethanol and 100% ACN. Proteins were digested first with Lys-C (enzyme-to-protein ratio, 1:50) in 50 mM HEPES (pH 7.6) containing 10% ACN for 3 h at 37 °C, followed by trypsin digestion (enzyme-to-protein ratio, 1:50) in the same buffer for 14 h at 37 °C with gentle shaking. Peptides were collected, quantified, subjected to an additional SP3 clean-up, and dried by vacuum centrifugation.

Approximately 55 μg peptide per sample was resuspended in 100 mM triethylammonium bicarbonate (TEAB), pH 8.5, and labeled with TMTpro reagents (Thermo Fisher Scientific) according to the manufacturer’s instructions. Labeling efficiency was evaluated by LC–MS/MS before samples were pooled. The pooled samples were purified by solid-phase extraction using Strata-X-C cartridges (Phenomenex) and dried by vacuum centrifugation.

High-resolution isoelectric focusing (HiRIEF) was performed as previously described^31^. TMT-labeled peptide pools were fractionated by immobilized pH gradient isoelectric focusing (IPG-IEF) over a pI range of 3–10. Dried peptides were dissolved in 250 μL rehydration solution containing 8 M urea and loaded onto 24-cm linear-gradient IPG strips (GE Healthcare), which were rehydrated overnight in 8 M urea containing 1% IPG Pharmalyte, pH 3–10. Following focusing, peptides were passively eluted into 72 contiguous fractions using sequential Milli-Q water, 35% ACN, and 35% ACN containing 0.1% formic acid with an in-house IPG extraction robot. Fractions were collected into 96-well plates, dried by vacuum centrifugation, and stored at −20 °C until LC–MS/MS analysis.

Online LC–MS/MS was performed as previously described^31^ using an UltiMate 3000 RSLCnano system coupled to a Q Exactive HF mass spectrometer (Thermo Fisher Scientific). Each fraction was resuspended in 20 μL solvent A, and 10 μL was injected. Peptides were trapped on an Acclaim PepMap 100 C18 precolumn (75 μm × 2 cm, 5 μm, 100 Å) and separated on an EASY-Spray PepMap RSLC C18 analytical column (75 μm × 50 cm, 2 μm, 100 Å). Solvent A consisted of 95% water, 5% DMSO, and 0.1% formic acid, and solvent B consisted of 5% water, 5% DMSO, 95% ACN, and 0.1% formic acid. Peptides were separated at a flow rate of 0.25 μL/min using fraction-specific gradients increasing from approximately 6%–8% B to 40% B, followed by an increase to 100% B.

Full MS scans were acquired at a resolution of 60,000 over an m/z range of 300–1500. Data-dependent MS/MS spectra were acquired for the five most abundant precursor ions at a resolution of 30,000 using higher-energy collisional dissociation (HCD) with a normalized collision energy of 30%. Precursors were isolated using a 2 m/z isolation window. Automatic gain control targets were 1 × 10⁶ for MS1 and 1 × 10⁵ for MS2, with maximum injection times of 100 ms for both MS1 and MS2. Dynamic exclusion was set to 30 s, and precursors with unassigned charge states or a charge state of 1 were excluded.

Protein identification and quantification were performed using MS-GF+ and Percolator within the Galaxy platform against the Ensembl Homo sapiens protein database (ENS109, February 2023 release). MSGF+ settings included precursor mass tolerance of 10 ppm, fully-tryptic peptides, maximum peptide length of 50 amino acids, and a maximum charge of 6. Fixed modifications were TMT on lysines and peptide N-termini, and carbamidomethylation on cysteine residues; a variable modification was used for oxidation on methionine residues. Quantification of TMT reporter ions was done using the OpenMS project’s IsobaricAnalyzer (v2.0)^52^. PSMs found at 1% FDR (false discovery rate) were used to infer gene identities.

Protein quantification by TMT reporter ions was calculated using TMT PSM ratios to the entire sample set and normalized to the sample median. The median PSM TMT reporter ratio from peptides unique to a gene symbol was used for quantification. Protein FDRs were calculated using the picked-FDR method using gene symbols as protein groups and limited to 1% FDR. Differential protein abundance analysis was performed using DEqMS^53^.

### Phosphoproteomics

Phosphoproteomic sample preparation was performed as previously described^54^, with cell treatment, protein extraction, SP3 clean-up, digestion, and TMT labeling performed similarly to the quantitative proteomics workflow except where indicated below.

Cells were adjusted to 1 × 10⁶ cells/mL and treated with 1 μM TCN (*n* = 3 replicates per time point). Untreated samples were collected at 0 min, followed by the collection of TCN-treated samples at 15 min, 45 min, 1.5 h, 3 h, and 6 h. Cells were collected by centrifugation at 500 × *g* for 3 min and washed twice with calcium-, magnesium-, and phenol red-free HBSS. Cell pellets were immediately lysed in 200 μL buffer containing 4% SDS, 25 mM HEPES (pH 7.6), 1 mM DTT, and Halt Protease and Phosphatase Inhibitor Cocktail (Thermo Fisher Scientific). Lysates were heated at 95 °C for 5 min and stored at −80 °C until further processing.

SP3 protein clean-up, enzymatic digestion, and TMT labeling were performed as described above for quantitative proteomics. TMT-pooled samples were subsequently purified using Strata-X reversed-phase cartridges (Phenomenex).

Peptides were fractionated by high-pH reversed-phase chromatography using an Agilent 1200 HPLC system with a 96-min gradient. Ninety-six fractions of 200 μL were collected and concatenated into 13 fractions, which were dried prior to phosphopeptide enrichment.

Phosphopeptides were enriched using Ti-IMAC HP magnetic beads (ReSyn Biosciences). Dried fractions were resuspended in binding buffer containing 80% ACN, 5% trifluoroacetic acid (TFA), and 0.5 M lactic acid. Ti-IMAC beads were equilibrated in binding buffer and incubated with the peptide samples for 30 min at 37 °C with shaking. Beads were washed with 80% ACN containing 0.1% TFA and subsequently with binding buffer. Bound phosphopeptides were eluted using 1% ammonium hydroxide, immediately acidified with formic acid, and dried prior to LC–MS/MS analysis.

Protein and phosphopeptide identification and quantification were performed using MS-GF+ and Percolator within the Galaxy platform against the UniProt Homo sapiens reference proteome, including isoforms (UniProt release 2023_03) or using FragPipe (version 23.1) against the UniProt Homo sapiens reference proteome (UP000005640, downloaded on 27 August 2025).

### Proteome integral solubility alteration assay

PISA sample preparation was performed as described previously^30^, with modifications. ALL-SIL cell lysates were treated with DMSO, TCN-P (1 or 100 μM), or TCN (100 μM), with four biological replicates per condition. Following compound exposure for 10 min at room temperature, lysates were subjected to a thermal challenge across 12 temperature points ranging from 43.0 to 61.7 °C in 1.7 °C increments for 3 min. Samples were subsequently cooled at room temperature for 3 min, and lysates from the 12 temperature points corresponding to each replicate were pooled. Aggregated proteins were removed by centrifugation at 20,000 × *g* for 30 min at 4 °C, and the soluble protein fractions were collected for proteomic analysis.

Proteins were digested using RapiZyme Trypsin (Waters), and peptides were processed using a modified SP3 clean-up and digestion protocol^51^. Peptides were labeled with TMTpro reagents (Thermo Fisher Scientific) and fractionated by IPG-IEF using pH 3–10 and pH 3.7–4.5 strips according to the HiRIEF method^31^.

Peptide fractions were analyzed using an UltiMate 3000 RSLCnano system coupled online to an Orbitrap Exploris 480 mass spectrometer (Thermo Fisher Scientific). Samples were trapped on an Acclaim PepMap 100 C18 precolumn (75 μm × 2 cm, 5 μm, 100 Å) and separated on a 50-cm EASY-Spray PepMap RSLC C18 analytical column (75 μm × 50 cm, 2 μm, 100 Å) at a flow rate of 0.25 μL/min. Full MS scans were acquired at a resolution of 60,000 over an m/z range of 375–1375, followed by data-dependent MS/MS of the top five precursor ions at a resolution of 30,000 using HCD with a normalized collision energy of 30%. Precursors were isolated using a 2 m/z window. AGC targets were 1 × 10⁶ for MS1 and 1 × 10⁵ for MS2, with maximum injection times of 100 ms for both. Dynamic exclusion was set to 30 s, and precursors with unassigned charge states or a charge state of 1 were excluded.

Protein identification and quantification were performed using MS-GF+ and Percolator within the Galaxy platform against the Ensembl Homo sapiens protein database (ENS111, January 2024 release). Differential protein solubility analysis was performed using DEqMS^53^.

### Quantification of TCN and TCN-P in cell extracts and media

Intracellular and extracellular concentrations of TCN and TCN-P were quantified by LC–MS/MS.

For intracellular metabolite analysis, 5 × 10⁶ cells were collected and extracted in 500 μL of ice-cold 90% methanol. Samples were disrupted by bead-based homogenization using a tungsten bead (30 kHz, 2 min), followed by centrifugation at 14,000 × *g* for 10 min at 4 °C. A total of 400 μL of the cleared supernatant was transferred to a 96-well plate and dried under a nitrogen stream. Dried extracts were reconstituted in 100 μL of 0.1% formic acid in water, and 0.5 μL of each sample was injected for LC–MS/MS analysis.

For extracellular measurements, 50 μL of culture medium was mixed with 450 μL of 100% methanol and vortexed for 2 min to precipitate proteins. Samples were centrifuged at 14,000 × g for 10 min at 4 °C, and 400 μL of the supernatant was transferred to a 96-well plate and dried under nitrogen. Samples were reconstituted in 100 μL of 0.1% formic acid in water, and 1 μL was injected for LC–MS/MS analysis.

Quantitative analysis was performed using a triple quadrupole mass spectrometer (Agilent 6495D) operated in multiple reaction monitoring (MRM) mode. Peak integration and quantification were performed using Mass Hunter Quantitative Analysis (version 12.0, Agilent). Intracellular and extracellular concentrations of TCN and TCN-P were calculated based on external calibration curves ranging from 10 pM to 100 µM for both compounds.

Three intracellular TCN-P measurements (one from the P12-ICHIKAWA 1 h group, one from the P12-ICHIKAWA 2 h group, and one from the ALL-SIL 24 h group) were identified as outliers using the IQR method (1.5× IQR criterion) and excluded from the plotted dataset. They were considered technical outliers based on the IQR criterion and inconsistent with the remaining replicate measurements within the corresponding time-point group.

### Experimental design and statistical analysis

All proteomics experiments were performed with at least three biological replicates. Statistical analyses were conducted using one-way ANOVA or a moderated *t*-test as appropriate. Differential protein abundance was calculated using DEqMS^53,55^, and differentially expressed proteins were defined using the following thresholds: ALL-SIL, |log₂FC | > 0.5 and adjusted *p*-value < 0.01; KASUMI-10, |log₂FC | > 0.38 and adjusted *p*-value < 0.01; P12-ICHIKAWA and KASUMI-2, |log₂FC | > 0.38 and adjusted *p*-value < 0.05. Phosphoproteomic comparisons were performed using limma^56^. The *p*-values were adjusted for multiple testing using the Benjamini-Hochberg method unless otherwise stated.

### Databases and bioinformatic analyses

Drug sensitivity (DSRT) data were obtained from the FORALL database^33^. PRISM drug repurposing data were obtained from DepMap^34^. UMAP data were downloaded on 10 October 2025. The TCN-P PRISM Repurposing Primary (Viability) dataset was downloaded on 20 May 2026, and TCN-P viability data together with *ADK* mRNA expression data [log2(TPM + 1)] were downloaded on 9 June 2026. The TCN and ADK inhibitor ABT-702 combination killing plot was generated using Synergyfinder^57^. Kaplan-Meier survival analysis of AML patients was obtained from the KM plotter^46^. GSEA and ssGSEA analyses were performed using msigdbr and GSVA^58–60^. Pathway enrichment networks were visualized using aPEAR^61^, and kinase activity inference was performed using KSEA^62^. Figures were created using BioRender, GraphPad Prism, and Adobe Illustrator.

## Supplementary information

Supplementary Information

## Data Availability

The mass spectrometry proteomics data have been deposited to the ProteomeXchange Consortium via the PRIDE^63^ partner repository with the dataset identifier PXD078136.

## Code availability

The R analysis code is available on GitHub (https://github.com/XueKang-Qi-1024/triciribine_leukemia).
