# Supplementary material for: Multi-omics profiling identifies triciribine as a biomarker-guided antimetabolite therapy for acute lymphoblastic leukemia
Source: NPJ Precis Oncol. 2026 Oct 14;10:390. doi: 10.1038/s41698-026-01736-z (PMC13669360; doi:10.1038/s41698-026-01736-z)
Supplement: Supplementary file 1 [file 41698_2026_1736_MOESM1_ESM.pdf]

Supplementary figures 1-6 related to:

Multi-omics profiling identifies triciribine as a biomarker-guided antimetabolite therapy for acute lymphoblastic leukemia

Authors

Xuekang Qi[1*], Georgios Mermelekas[1], Ondrej Hodek[2], Luay Aswad[1], Quentin Glaziou[1], Isabelle Rose Leo[1], Nona Struyf[3], Femke M. Hormann[4], Ioannis Siavelis[1], Henri Colyn Bwanika[5], Owen F.J. Hovey[1], Martin Haraldsson[6], Sean G. Rudd[4], Annika I. Johansson[7], Tom Erkers[3], Katja Pokrovskaja Tamm[5], Janne Lehtiö[1], Rozbeh Jafari[1*]

Affiliations

[1] Clinical Proteomics Mass Spectrometry, Department of Oncology-Pathology, Karolinska Institutet, Science for Life Laboratory, Tomtebodavägen 23A, 171 65 Solna, Sweden

[2] Swedish Metabolomics Centre, Department of Forest Genetics and Plant Physiology, Swedish University of Agricultural Sciences, Umeå, Sweden

[3] Molecular Precision Medicine, Department of Oncology-Pathology, Karolinska Institutet, Science for Life Laboratory, Tomtebodavägen 23A, 171 65 Solna, Sweden

[4] Department of Oncology-Pathology, Karolinska Institutet, Science for Life Laboratory, Tomtebodavägen 23A, 171 65 Solna, Sweden

[5] Department of Oncology-Pathology, Karolinska Institutet, J6:140 BioClinicum, Akademiska stråket 1, 171 64 Solna, Sweden

[6] Chemical Biology Consortium Sweden, Science for Life Laboratory, Department of Medical Biochemistry and Biophysics, Karolinska Institutet, 171 65 Stockholm, Sweden

[7] Swedish Metabolomics Centre, Department of Plant Physiology, Umeå University, Umeå, Sweden

* Corresponding authors

Correspondence should be addressed to: Xuekang Qi (xuekang.qi@ki.se) and Rozbeh Jafari (rozbeh.jafari@ki.se)

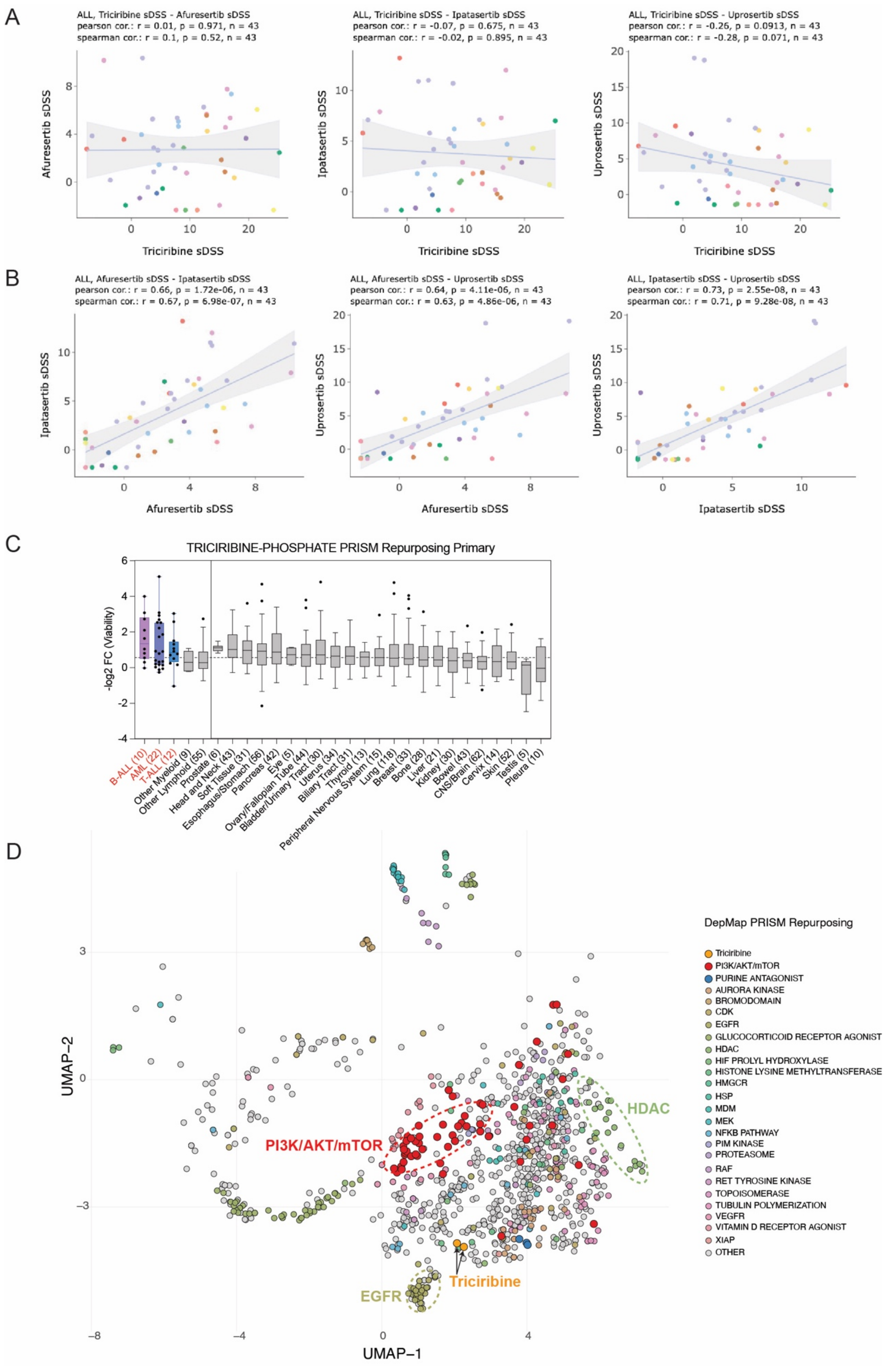
A
ALL, Triciribine sDSS - Afuresertib sDSS
pearson cor.: r = 0.01, p = 0.971, n = 43
spearman cor.: r = 0.1, p = 0.52, n = 43
Afuresertib sDSS
Triciribine sDSS
ALL, Triciribine sDSS - Ipatasertib sDSS
pearson cor.: r = -0.07, p = 0.675, n = 43
spearman cor.: r = -0.02, p = 0.895, n = 43
Ipatasertib sDSS
Triciribine sDSS
ALL, Triciribine sDSS - Uprosertib sDSS
pearson cor.: r = -0.26, p = 0.0913, n = 43
spearman cor.: r = -0.28, p = 0.071, n = 43
Uprosertib sDSS
Triciribine sDSS
B
ALL, Afuresertib sDSS - Ipatasertib sDSS
pearson cor.: r = 0.66, p = 1.72e-06, n = 43
spearman cor.: r = 0.67, p = 6.98e-07, n = 43
Ipatasertib sDSS
Afuresertib sDSS
ALL, Afuresertib sDSS - Uprosertib sDSS
pearson cor.: r = 0.64, p = 4.11e-06, n = 43
spearman cor.: r = 0.63, p = 4.86e-06, n = 43
Uprosertib sDSS
Afuresertib sDSS
ALL, Ipatasertib sDSS - Uprosertib sDSS
pearson cor.: r = 0.73, p = 2.55e-08, n = 43
spearman cor.: r = 0.71, p = 9.28e-08, n = 43
Uprosertib sDSS
Ipatasertib sDSS
C
TRICIRIBINE-PHOSPHATE PRISM Repurposing Primary
-log2 FC (Viability)
B-ALL (10)
AML (22)
T-ALL (12)
Other Myeloid (9)
Other Lymphoid (55)
Prostate (6)
Head and Neck (43)
Soft Tissue (31)
Esophagus/Stomach (56)
Pancreas (42)
Eye (5)
Ovary/Fallopian Tube (44)
Bladder/Urinary Tract (30)
Uterus (34)
Biliary Tract (31)
Thyroid (13)
Peripheral Nervous System (15)
Lung (118)
Breast (33)
Bone (28)
Liver (21)
Kidney (30)
Bowel (43)
CNS/Brain (62)
Cervix (14)
Skin (52)
Testis (5)
Pleura (10)
D
UMAP−2
UMAP−1
PI3K/AKT/mTOR
HDAC
EGFR
Triciribine
DepMap PRISM Repurposing
Triciribine
PI3K/AKT/mTOR
PURINE ANTAGONIST
AURORA KINASE
BROMODOMAIN
CDK
EGFR
GLUCOCORTICOID RECEPTOR AGONIST
HDAC
HIF PROLYL HYDROXYLASE
HISTONE LYSINE METHYLTRANSFERASE
HMGCR
HSP
MDM
MEK
NFKB PATHWAY
PIM KINASE
PROTEASOME
RAF
RET TYROSINE KINASE
TOPOISOMERASE
TUBULIN POLYMERIZATION
VEGFR
VITAMIN D RECEPTOR AGONIST
XIAP
OTHER

Supplementary Figure 1. Triciribine exhibits a distinct drug sensitivity profile compared with Akt inhibitors.

A, Pairwise correlation analysis comparing the drug sensitivity profile of TCN with afuresertib, ipatasertib, and uprosertib across 43 ALL cell lines. Data were obtained from the FORALL database.

B, Pairwise correlation analysis of drug sensitivity profiles among afuresertib, ipatasertib, and uprosertib across 43 ALL cell lines. Data were obtained from the FORALL database.

C, TCN-P sensitivity of cell lines in the DepMap PRISM repurposing dataset. Cell lines are grouped by tissue of origin. Only tissue groups containing ≥ 5 cell lines are shown, with numbers in parentheses indicating the number of cell lines per group. Data are presented as −log2 fold change (viability). Boxes indicate the median and interquartile range (25th–75th percentile), and whiskers are plotted according to the Tukey method; outliers are shown as dots. For hematologic malignancy cell lines, all individual data points are displayed and whiskers represent minimum-to-maximum values. The dashed line indicates the median value across the full dataset. The left panel shows hematologic malignancy cell lines.

D, Two-dimensional UMAP projection of drug sensitivity profiles from the PRISM repurposing dataset (n = 578 cell lines). Compounds are positioned based on cosine similarity of killing profiles, and compounds with shared annotated mechanisms of action are indicated by color.

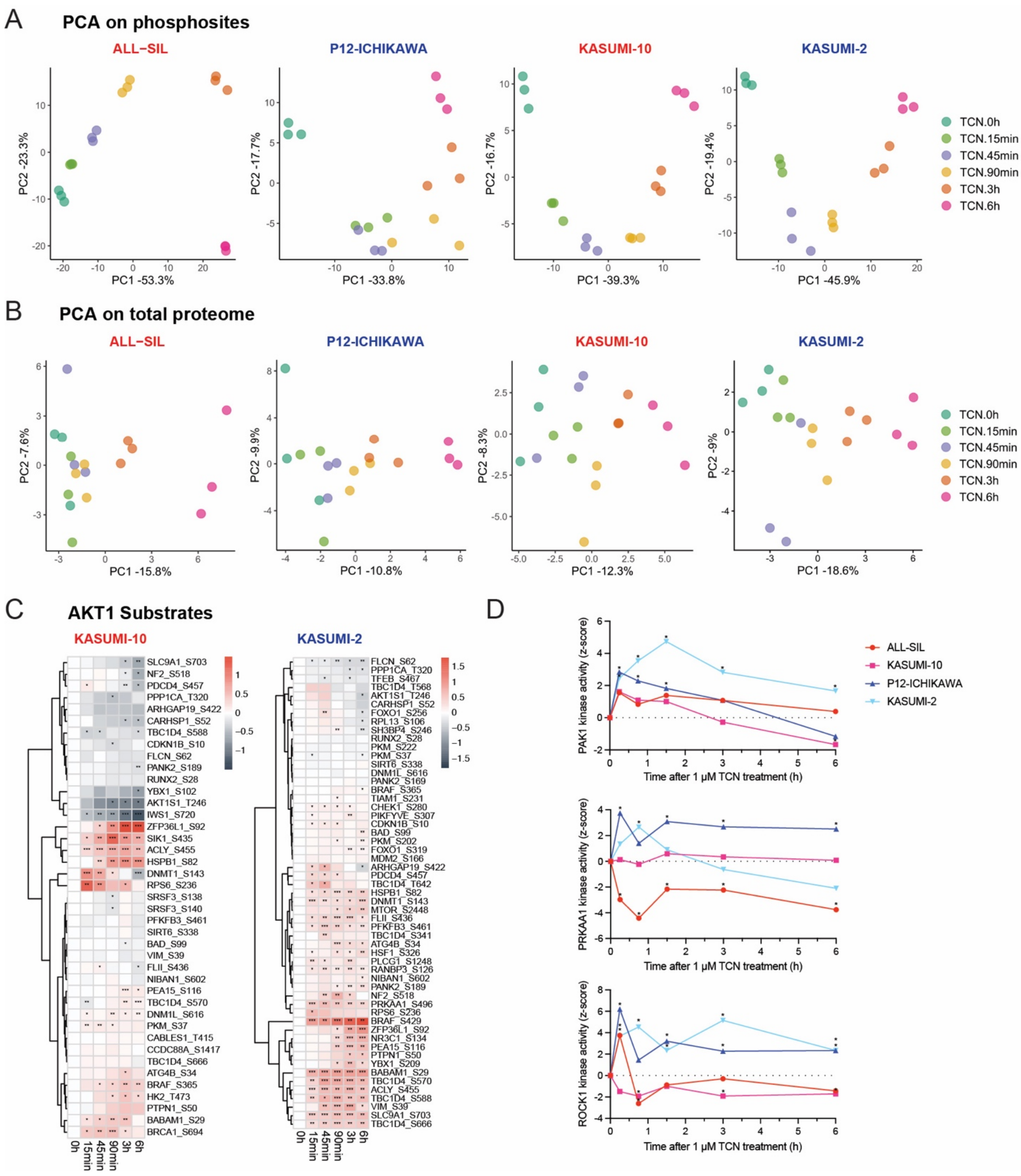

Supplementary Figure 2. Global phosphoproteomic and proteomic profiling following triciribine treatment.

A, Principal component analysis (PCA) of phosphosite-level quantitative data from four ALL cell lines (ALL-SIL, P12-ICHIKAWA, KASUMI-10, and KASUMI-2) treated with TCN across

indicated time points from 0 h to 6 h.

B, PCA of total proteome quantitative data from four ALL cell lines (ALL-SIL, P12-ICHIKAWA, KASUMI-10, and KASUMI-2).

C, Phosphorylation changes of known Akt1 substrates across time points relative to 0 h in KASUMI-10 and KASUMI-2 cells (n = 3 replicates per time point). Statistical comparisons were performed using two-tailed unpaired Welch's t-test where applicable. Statistical significance: * $p<0.05$; ** $p<0.01$, *** $p<0.001$.

D, Time-course changes in the inferred kinase activities of PAK1, PRKAA1 and ROCK1 following treatment with 1 μM TCN. KSEA z-scores are shown for each time point relative to the corresponding 0-h baseline within each ALL cell line. Statistical significance was determined by KSEA using a one-tailed test, followed by Benjamini–Hochberg correction for multiple testing. KSEA $p < 0.05$ is indicated by an asterisk.

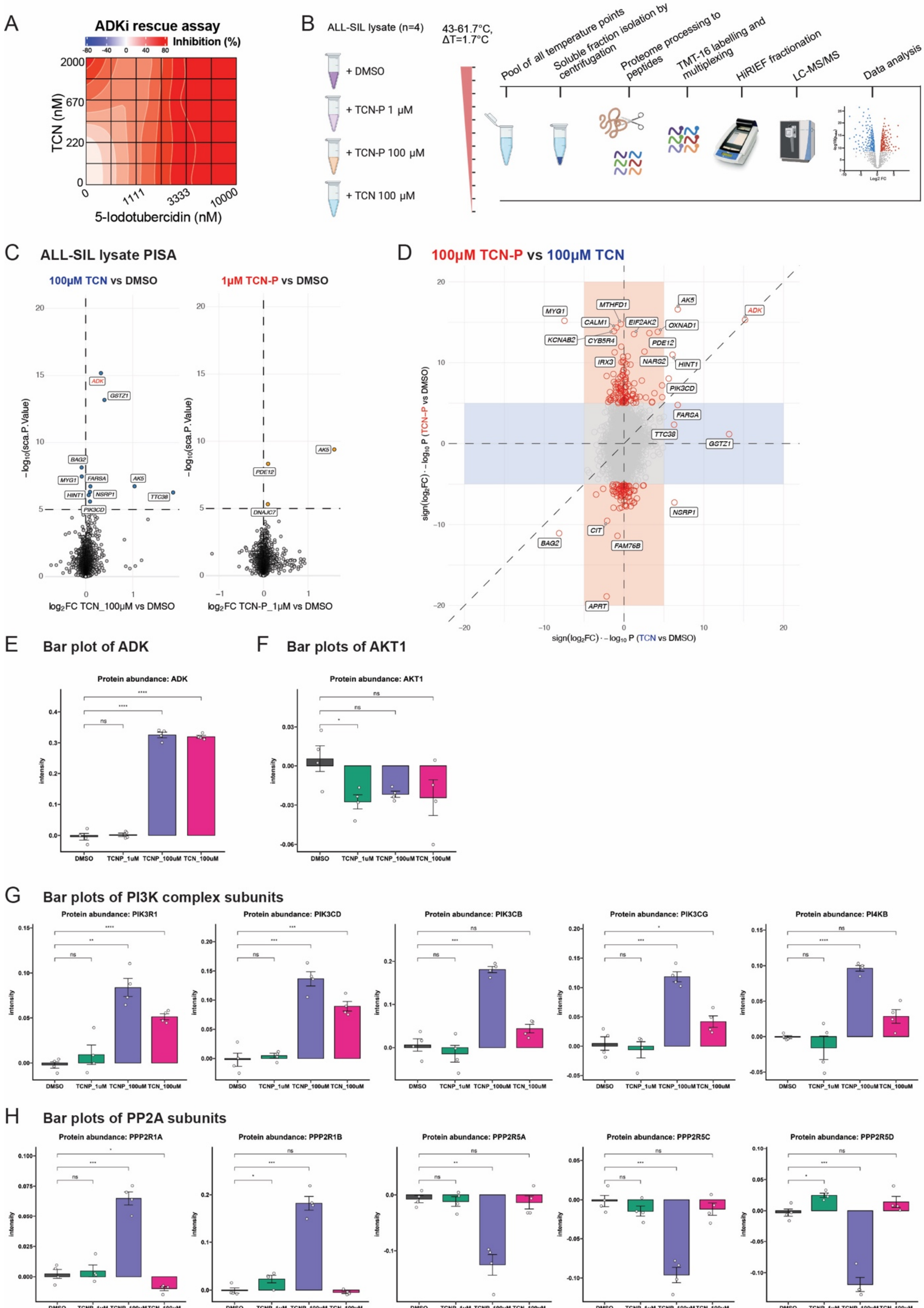

A
ADKi rescue assay
Inhibition (%)
TCN (nM)
5-Iodotubercidin (nM)
B
ALL-SIL lysate (n=4)
+ DMSO
+ TCN-P 1 µM
+ TCN-P 100 µM
+ TCN 100 µM
43-61.7°C, ΔT=1.7°C
Pool of all temperature points
Soluble fraction isolation by centrifugation
Proteome processing to peptides
TMT-16 labelling and multiplexing
HiRIEF fractionation
LC-MS/MS
Data analysis
C
ALL-SIL lysate PISA
100µM TCN vs DMSO
1µM TCN-P vs DMSO
−log10(sca.P.Value)
log2FC TCN_100µM vs DMSO
log2FC TCN-P_1µM vs DMSO
D
100µM TCN-P vs 100µM TCN
sign(log2FC) · −log10 P (TCN-P vs DMSO)
sign(log2FC) · −log10 P (TCN vs DMSO)
E
Bar plot of ADK
Protein abundance: ADK
F
Bar plots of AKT1
Protein abundance: AKT1
G
Bar plots of PI3K complex subunits
Protein abundance: PIK3R1
Protein abundance: PIK3CD
Protein abundance: PIK3CB
Protein abundance: PIK3CG
Protein abundance: PI4KB
H
Bar plots of PP2A subunits
Protein abundance: PPP2R1A
Protein abundance: PPP2R1B
Protein abundance: PPP2R5A
Protein abundance: PPP2R5C
Protein abundance: PPP2R5D
intensity
DMSO
TCNP_1uM
TCNP_100uM
TCN_100uM

Supplementary Figure 3. Expanded PISA analysis of candidate triciribine-interacting proteins.

A, Cell viability and cell number measurements in ALL-SIL cells treated with varying concentrations of TCN and the ADK inhibitor (ADKi) 5-Iodotubercidin, alone or in combination, for 3 days. Live cell numbers were quantified using the Via1 cassette–NucleoCounter and used as input for SynergyFinder to evaluate the rescue effect of 5-Iodotubercidin on TCN-induced cytotoxicity.

B, Experimental design and workflow of the Proteome Integral Solubility Alteration (PISA) assay.

C, Volcano plots of PISA results showing changes in protein solubility following treatment with 100 µM TCN or 1 µM TCN-P compared with DMSO control. Statistical analysis was performed using DEqMS, and DEqMS-derived p-values (sca.P.Value) are shown on the y-axis, and $\log_2$FC (fold change) on the x-axis.

D, Comparison of PISA results between 100 µM TCN-P and 100 µM TCN treatments. The x- and y-axes represent signed −log10(p-values) multiplied by the direction of solubility change ($\log_2$FC).

E–H, Bar plots showing solubility changes of selected candidate TCN-P–interacting proteins identified by PISA. Statistical comparisons were performed using two-tailed unpaired Welch's t-test where applicable. Statistical significance is indicated as * $p<0.05$; ** $p<0.01$, *** $p<0.001$.

A **ALL−SIL TCN 24h: GSEA**

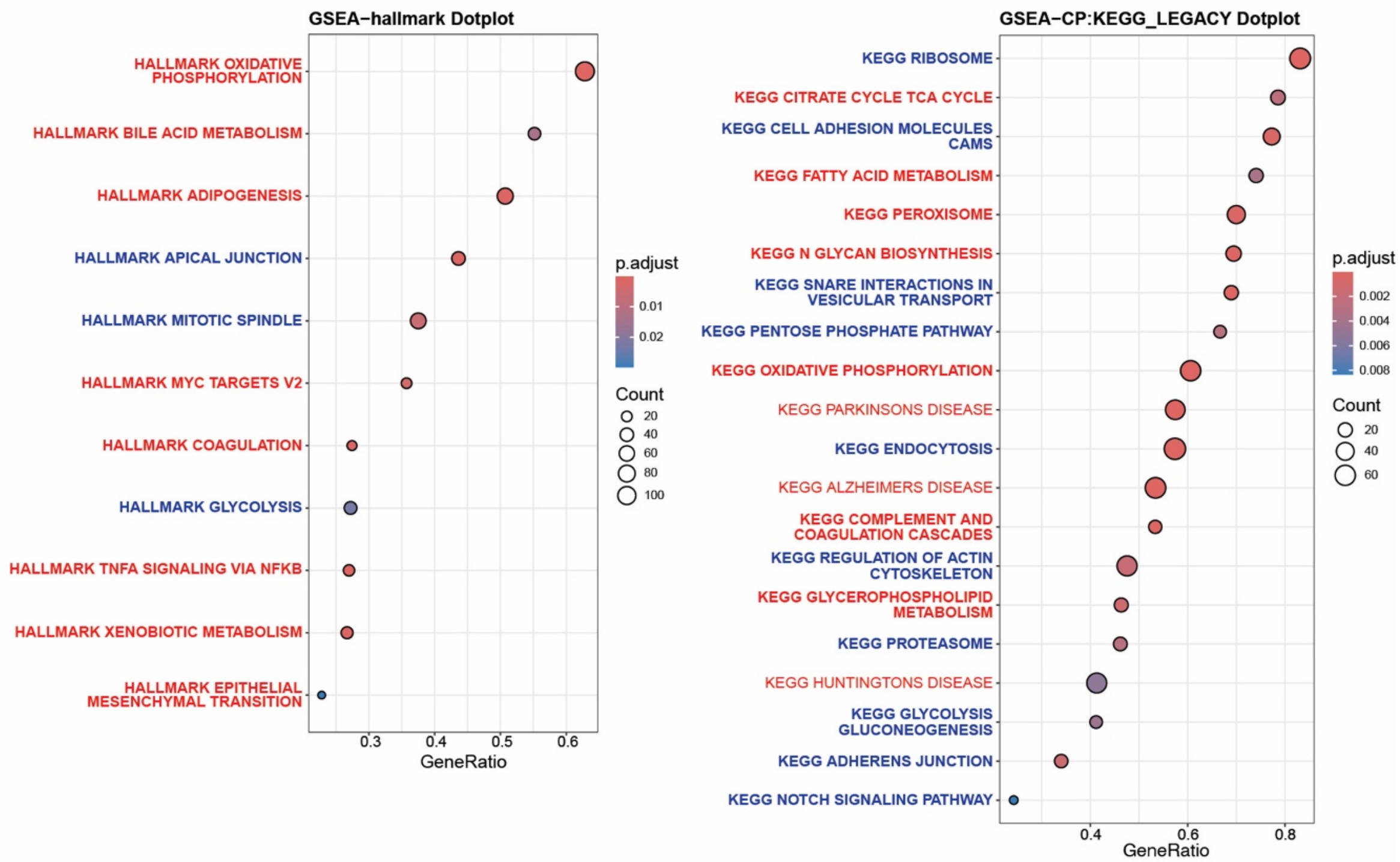

B **ALL−SIL TCN 24h: GO BP up-regulation**

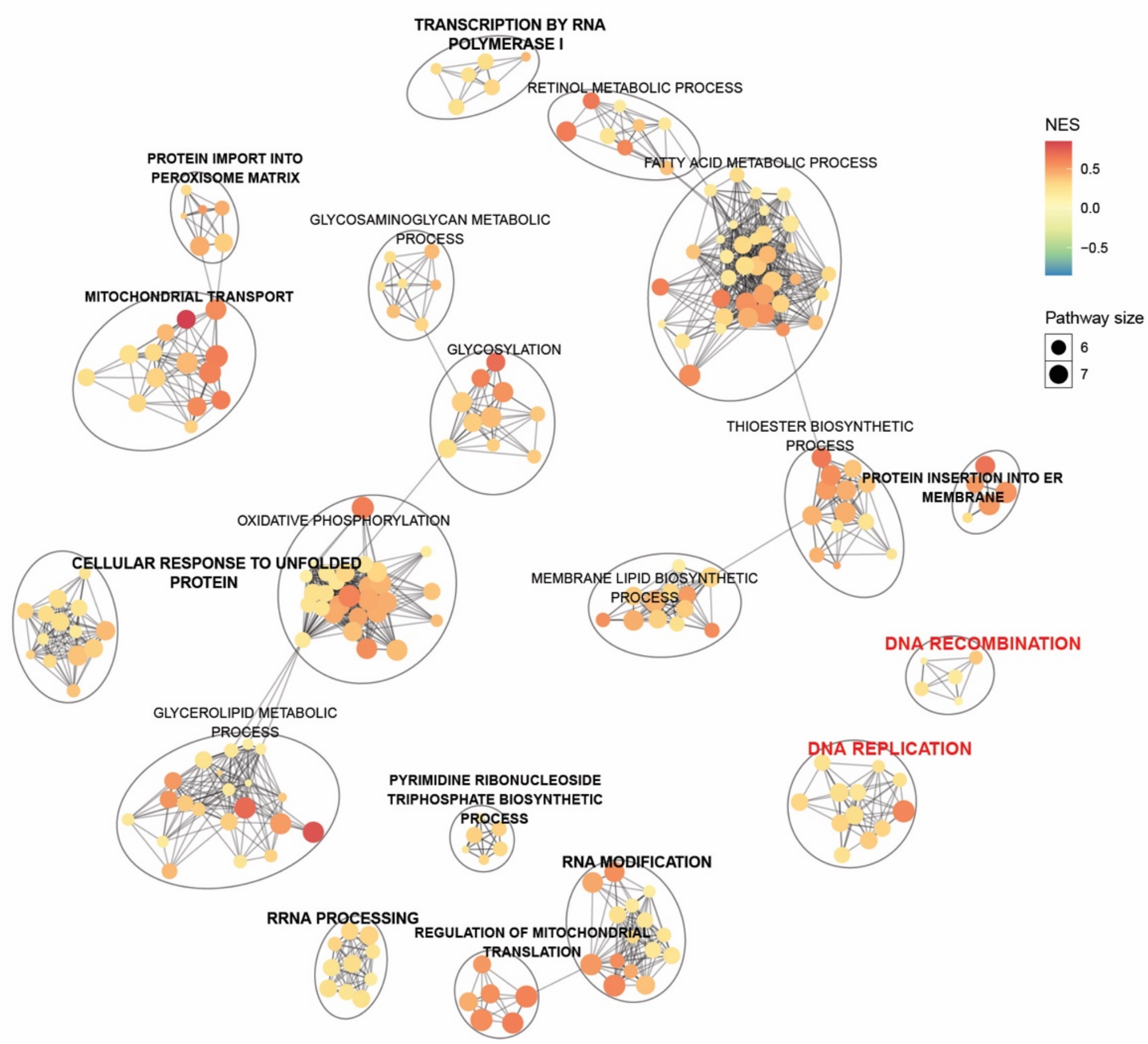

Supplementary Figure 4. Pathway-level proteomic responses.

A, Dot plot summarizing gene set enrichment analysis results using Hallmark and KEGG databases for ALL-SIL cells treated with TCN for 24 h compared with DMSO. Red text indicates pathways or signatures upregulated in TCN-treated cells relative to DMSO, whereas blue text indicates downregulated pathways.

B, aPEAR visualization of significantly up-regulated biological process networks identified by ssGSEA in ALL-SIL cells treated with TCN for 24 h compared with DMSO.

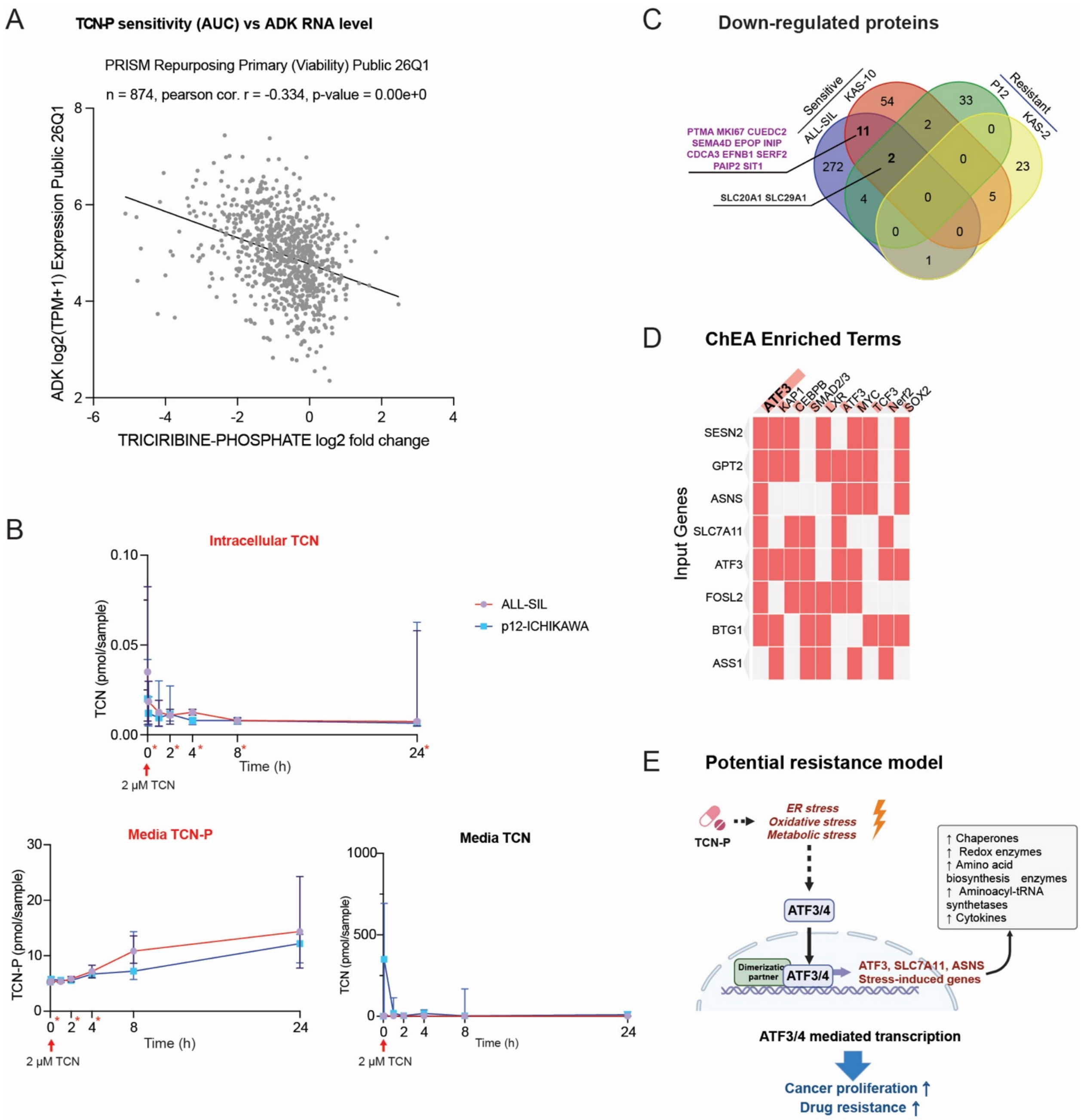

Supplementary Figure 5. ADK expression, triciribine metabolism, and comparative proteomic responses.

A, Correlation between ADK mRNA expression and TCN-P sensitivity in the PRISM Repurposing Primary cohort.

B, Time-course quantification of intracellular TCN, extracellular TCN-P, and extracellular TCN concentrations following treatment with 2 μM TCN in ALL-SIL and P12-ICHIKAWA cells. n = 6 replicates were analyzed for each time point. Intracellular TCN and extracellular TCN-P

concentrations at time points labeled with * were low in abundance and close to blank/background measurements.

C, Venn diagram showing the overlap of proteins significantly downregulated by TCN treatment at both 6 h and 24 h across four ALL cell lines (ALL-SIL, P12-ICHIKAWA [P12], KASUMI-10 [KAS-10], and KASUMI-2 [KAS-2]).

D, ChEA analysis identifying candidate transcription factors associated with proteins upregulated in less-sensitive ALL cell lines P12-ICHIKAWA and KASUMI-2 following TCN treatment.

E, Schematic representation of a proposed model summarizing cellular features associated with reduced TCN sensitivity in ALL cell lines.

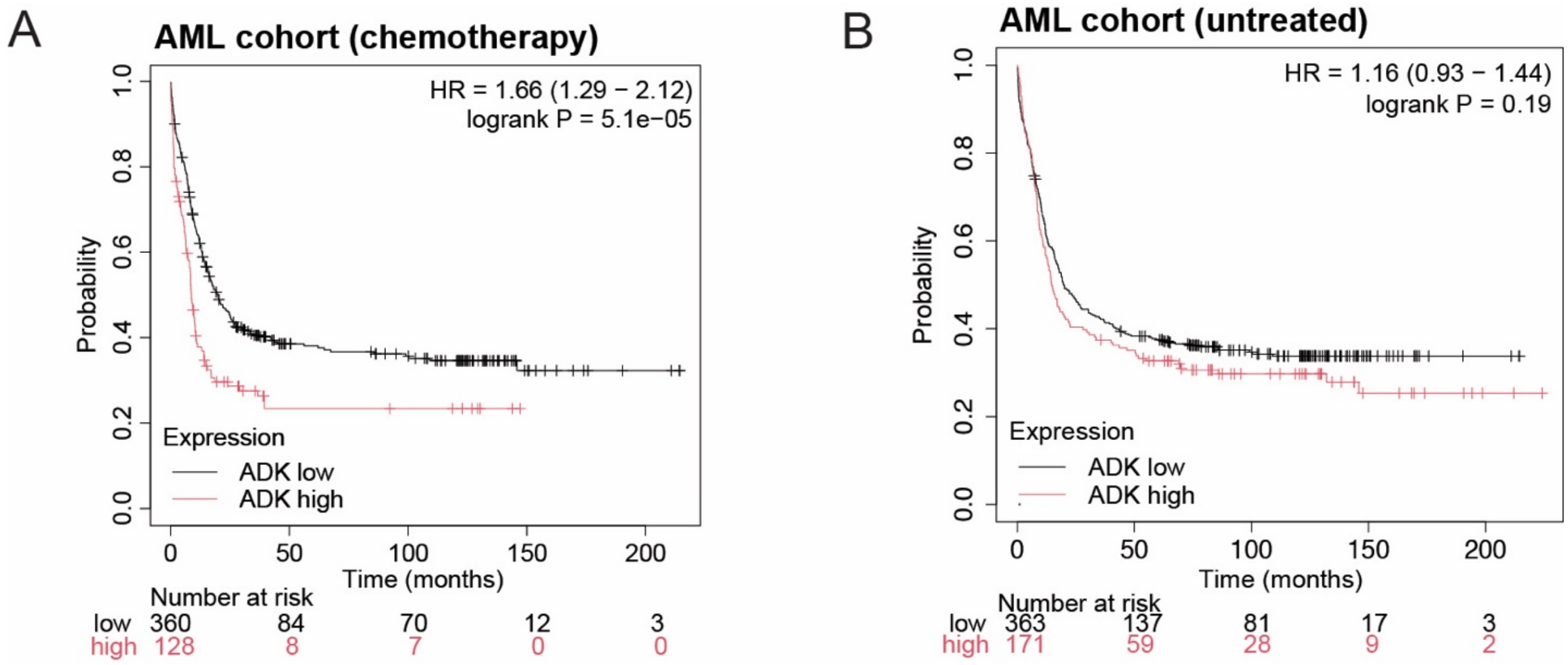

Supplementary Figure 6. A-B, Kaplan-Meier survival analysis of chemotherapy-treated or untreated AML patients stratified by ADK mRNA levels. Data were obtained from the KM plotter platform. The hazard ratio (HR) with 95% confidence intervals and logrank P value are shown in the plots.
